# Iron–Manganese Dual-Doping Tailors the Electronic Structure of Na_3_V_2_(PO_4_)_2_F_3_ for High-Performance Sodium-Ion Batteries

**DOI:** 10.1007/s40820-025-01881-3

**Published:** 2026-01-05

**Authors:** Jien Li, Shuang Luo, Renjie Li, Yingkai Hua, Linlong Lyu, Xiangjun Pu, Jun Fan, Zheng-Long Xu

**Affiliations:** 1 Department of Industrial and Systems Engineering, The Hong Kong Polytechnic University, Hung Hom, Hong Kong, 999077 People’s Republic of China; 2 Department of Materials Science and Engineering, City University of Hong Kong, Hong Kong, 999077 People’s Republic of China; 3 Research Institute for Advanced Manufacturing, The Hong Kong Polytechnic University, Hung Hom, Hong Kong, 999077 People’s Republic of China; 4 School of Resources, Environment and Materials, Guangxi University, Nanning, 530004 People’s Republic of China

**Keywords:** Sodium-ion batteries, Sodium fluorophosphates, Electronic structure, Fe–Mn co-doping

## Abstract

Sodium superionic conductor (NASICON)-type materials are promising cathodes for sodium-ion batteries due to their stable multi-channel frameworks and exceptional ionic conductivity. Among them, Na_3_V_2_(PO_4_)_2_F_3_ (NVPF) has attracted significant attention. However, the low electronic conductivity and phase impurities limit its sodium storage capability. Herein, we present a Fe and Mn dual-doped NVPF (FM-NVPF) cathode with improved phase purity, electronic conductivity, and electrochemical activities. Detailed ex-situ analyses and density functional theory calculations reveal that Fe and Mn dopants induce defect energy levels and modulate the electronic structure, resulting in a direct-to-indirect bandgap transition in NVPF, which in turn increases carrier concentration and lifetime, accelerates ionic/electronic transport, and improves structural stability. As a result, the FM-NVPF cathode delivers a high capacity of 126.6 mAh g⁻^1^ at 0.1 C (1 C = 128 mAh g⁻^1^) and outstanding high-rate capability of 67.6 mAh g⁻^1^ at 50 C, corresponding to 1.2 min per charge. Furthermore, Na ion full cells assembled with the FM-NVPF cathodes and hard carbon anodes exhibit a high energy density of about 175 Wh kg^−1^_cathode+anode mass_ and appealing cyclic stability. This work provides an efficient strategy for developing high-purity and high-performance NVPF cathode materials for advanced sodium-ion batteries.

**Supplementary Information:**

The online version contains supplementary material available at 10.1007/s40820-025-01881-3.

## Highlights

- Regulation of the electronic structure of Na_3_V_2_(PO_4_)_2_F_3_ (NVPF) via iron–manganese dual-doping enhances electrical conductivity and ion diffusion kinetics.
- Efficient charge transport and highly reversible Na^+^ de/intercalation in Fe-Mn dual-doped NVPF (FM-NVPF) enable exceptional rate capability and charge storage capacity.
- The full cell with the FM-NVPF cathode and hard carbon anode displays superior rate performance and cycling stability.

**Supplementary Information:**

The online version contains supplementary material available at 10.1007/s40820-025-01881-3.

## Graphical abstract

**Figure:**
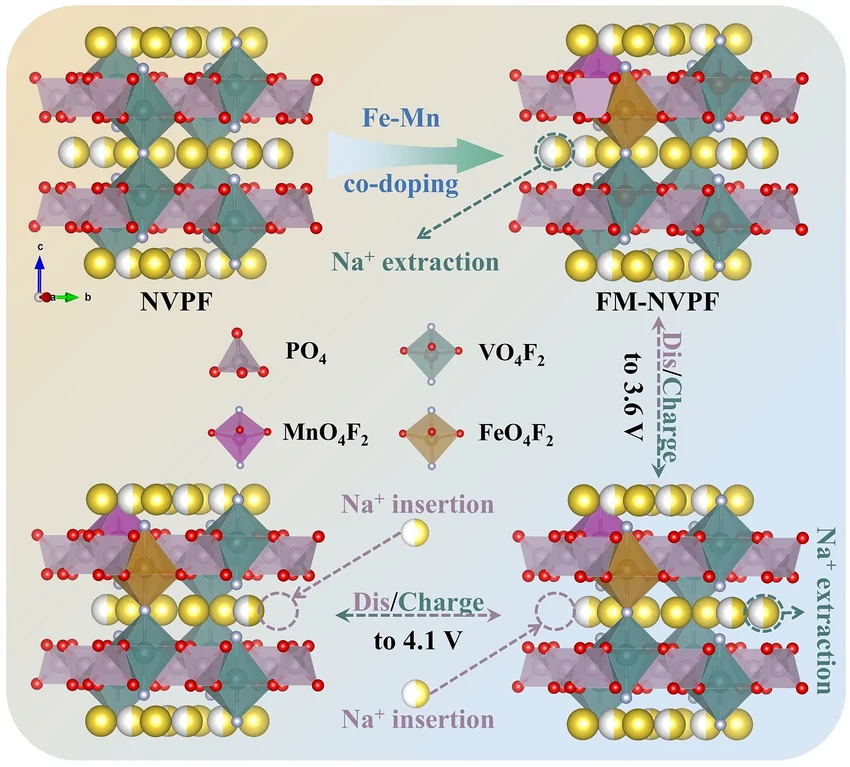

## Introduction

With the increasing demand for sustainable energy storage solutions, sodium-ion batteries (SIBs) have emerged as promising alternatives to lithium-ion batteries due to the abundant and low-cost availability of sodium resources [1–3]. However, the development of SIBs remains challenged by the sluggish kinetics and limited cycling stability of available cathode materials [4, 5]. Cathode materials for SIBs mainly include layered transition metal oxides, polyanionic compounds, and Prussian blue analogs, among which polyanionic structures with three-dimensional ion conduction channels and rigid frameworks are specifically promising for long-life SIBs [6]. Na_3_V_2_(PO_4_)_2_F_3_ (NVPF) emerges as a particularly promising candidate in this category, featuring a three-dimensional framework of corner-sharing VO_6_ octahedra and PO_4_ tetrahedra that form interconnected channels for rapid Na^+^ diffusion [7]. While NVPF exhibits attractive characteristics including a high theoretical capacity (128 mAh g⁻^1^), appropriate redox potentials, and robust structural stability, its practical implementation is hindered by poor electronic conductivity and phase impurity formation during synthesis, which collectively degrade rate capability and cycling durability [8, 9]. 

To mitigate these obstacles, strategies such as carbon coating, nanosizing and transition metal doping have been employed to enhance the electronic conductivities, reduce the ion diffusion length, and regulate the electronic structures [10–12]. Among them, transition metal doping at the vanadium redox center serves multiple functions, including improving electronic conductivity, reducing Na ion diffusion energy barriers, and strengthening structural stability [13]. Affordable iron (Fe) and manganese (Mn) have attracted attention as dopants in NVPF cathode materials. For example, Park et al*.* prepared Fe-doped NVPF/Na_3_V_2_(PO_4_)_2_ (NVP) with rational Fe contents, which showed optimal performance of 118 mA h g^−1^ at 0.5 C under Fe-doping content of 8.9 at% [14]. Li et al*.* investigated the impact of Fe-dopant stoichiometry on the volcanic electronic conductivity and electron activation energy for Na_3_V_2-2x_Fe_2x_(PO_4_)_2_F_3_, revealing a transition in the electron charge transfer process converts from V to Fe ions at *x* = 0.03 [15]. Zhang et al*.* utilized the polyol-assisted hydrothermal synthesis and chemical vapor deposition method to prepare Na_3_V_1.95_Mn_0.05_(PO_4_)_2_F_3_@C hollow microspheres, where the Mn^2+^ doping and carbon coating improved the electrical conductivity and Na ion diffusion coefficient [16]. Despite the successful integration of Fe or Mn into the NVPF lattice, the doped cathode materials often exhibit a certain amount of NVP impurities [17, 18], and the effects of Fe and Mn co-doping on NVPF structure and performance remain unexplored.

Herein, we successfully developed phase-pure Fe–Mn co-doped NVPF (FM-NVPF), where the dual-cation co-doping strategy effectively regulates crystal nucleation dynamics. This dual-doping approach offers synergistic effects in controlling NVPF phase purity and modulating the electronic structures of NVPF, which are absent for the Fe-doped, Mn-doped, or pristine NVPF samples. The combined experimental and theoretical analyses conclusively demonstrate that the FM-NVPF exhibits superior charge transfer kinetics compared to pristine NVPF. In Na ion half cells, the FM-NVPF cathodes show excellent charge storage capability (126.6 mAh g^−1^ at 0.1 C), rate performance (67.5 mAh g^−1^ at 50 C), and long-term cycle stability (81.5% capacity retention after 1000 cycles at 0.5 C). Finally, the practical feasibility of FM-NVPF cathodes is demonstrated in the hard carbon//FM-NVPF Na ion full cells. This work provides an effective design paradigm for high-performance NASICON-type cathodes, offering new insights into the development of advanced sodium-ion batteries.

## Experimental Section

### Materials Synthesis

The FM-NVPF cathode material is prepared by using the sol-gel method, followed by the annealing treatment. All chemicals are utilized as purchase without further purifications. In specific, certain stoichiometric amounts of V_2_O_5_, Fe(NO_3_)_3_·9H_2_O, Mn(NO_3_)_2_·9H_2_O, NH_4_H_2_PO_4_, NaF, NH_4_F, H_2_C_2_O_4_·2H_2_O precursors are dissolved in 60 mL deionized water and magnetically stirred for about 1.5 h to obtain a uniform solution. Then, 1.5 mL NH_4_·H_2_O solution was added to adjust the pH value to 7. The solution was subsequently transferred to an oil bath at 80 °C and stirred overnight to evaporate the solvent. Finally, the dry powder was collected and annealed in a tube furnace at 600 °C for 4 h under flowing argon gas. The FM-NVPF sample was milled into powders for cathode preparation. In this process, NVPF with different doping amounts are prepared for comparison by adjusting the amounts of Fe and Mn reactants, named 0.5 FM (0.25 mmol Fe(NO_3_)_3_·9H_2_O + 0.25 mmol Mn(NO_3_)_2_·9H_2_O), FM (0.5 mmol + 0.5 mmol), 1.5 FM (0.75 mmol + 0.75 mmol) corresponding to the main research object (FM-NVPF), and 2 FM (1 mmol + 1 mmol). In addition, the samples of Fe-doped NVPF (denoted as F-NVPF), Mn-doped (denoted as M-NVPF), and NVPF without doping were also synthesized for comparison using the same way as control samples.

### Material Characterizations and Electrochemical Testing

The X-ray diffration (XRD) tests were conducted on a Rigaku SmartLab 9 kW diffractometer with Cu-Kα radiation. The Rietveld refinement was conducted through the GSAS program. The XPS test was performed on a Thermo Escalab 250Xi photoelectron spectrometer to evaluate the chemical structures. A Hitachi S-4800 field emission scanning electron microscope (SEM) and a JEOL JEM-2100F transmission electron microscope (TEM) were employed to examine the morphologies and microstructures of cathode materials. N_2_ adsorption and desorption were measured with an ASAP 2460 to test the pore structure of materials. Inductively coupled plasma (ICP, ICAP 7000) was used to quantitatively analyze the doping content of Fe, Mn element in the FM-NVPF. The band gap width of the materials was analyzed using UV–visible spectroscopy (UV–vis, Thermo ESCALAB 250XI). The electronic conductivity of materials was determined by the four-point probe (ST2742B-SD).

The cathodes were prepared by casting the uniform slurry containing 70 wt% active material (FM-NVP, F-NVPF, M-NVPF, or NVPF), 20 wt% carbon black, and 10 wt% polyvinylidene fluoride (PVDF) on an aluminum foil, followed by drying overnight under vacuum at 60 °C. The hard carbon anodes were synthesized by coating a slurry containing 70 wt% hard carbon material (HC), 20 wt% carbon black, and 10 wt% polyvinylidene fluoride (PVDF) on an aluminum foil, followed by the drying process.

For coin cell assembly, glass fiber membranes were employed as the separator, metallic Na as the counter and reference electrodes, 1 mol L^−1^ NaClO_4_ in propylene carbonate: ethylene carbonate (PC: EC = 1:1) with 5 vol% fluoroethylene carbonate (FEC) as the electrolyte. HC//FM-NVPF full cells were assembled with a negative: positive capacity ratio (N/P) of 1.1. The galvanostatic charge–discharge measurements were conducted on the Neware battery testers in the voltage window of 2.0–4.5 V versus Na^+^/Na at different current densities. Cyclic voltammetry (CV) was performed on a CHI 660E electrochemical workstation. The galvanostatic intermittent titration technique (GITT, Arbin battery tester) was performed to calculate the Na^+^ diffusion coefficients. Electrochemical impedance spectroscopy (EIS, Princeton Applied Research P2000) was performed to examine the charge transfer resistance (The frequency range of the in-situ EIS test is from 10 mHZ to 100 kHZ; the current density is 0.1 C; charging 28 segments and discharging 22 segments).

### Theoretical Calculations

The calculations were performed using density functional theory (DFT) and implemented through the Vienna *Ab*-*initio* Simulation Package (VASP) [19, 20]. The ion–electron interaction was represented using the projector augmented wave (PAW) method. At the same time, the electronic exchange–correlation function was treated with the generalized gradient approximation (GGA) proposed by Perdew–Burke–Ernzerhof (PBE) [21, 22]. The van der Waals (vdW) interactions were analyzed using Grimme’s DFT-D3 method [23]. The cutoff energy and energy convergence criteria were set to 500 and 10^–5^ eV, respectively. For geometric optimization, the k-point meshes of 2 × 2 × 2 and 1 × 1 × 1 were used for unit cells and supercells (2 × 2 × 1), respectively. For electronic property calculations, the density of states (DOS) was computed using the unit cell with a doubled k-point mesh [24]. Atomic positions were fully relaxed until the maximum force on each atom was less than 0.02 eV Å^−1^. Moreover, to clarify the dynamic diffusion characteristics of Na ions on the (001) surface of FM-NVPF and NVPF, the computational diffusion potential was determined through the climbing image nudged elastic band (CI-NEB) method [25].

The adsorption energies (*E*_ads_) of Na ions on the (001) surface of FM-NVPF and NVPF are calculated by Eq. 1:

1 $$E_{{{\mathrm{ads}}}} = E_{{{\mathrm{Na}}@{\mathrm{slab}}}} - E_{{{\mathrm{slab}}}} - E_{{{\mathrm{Na}}}}$$

where the *E*_Na@slab_, *E*_slab_, and *E*_Na_ represent the total energy with and without Na adsorbed on FM-NVPF, and a single atom from bulk structure, respectively.

## Results and Discussion

### The Preparation and Characterization of Materials

**Fig. 1:**
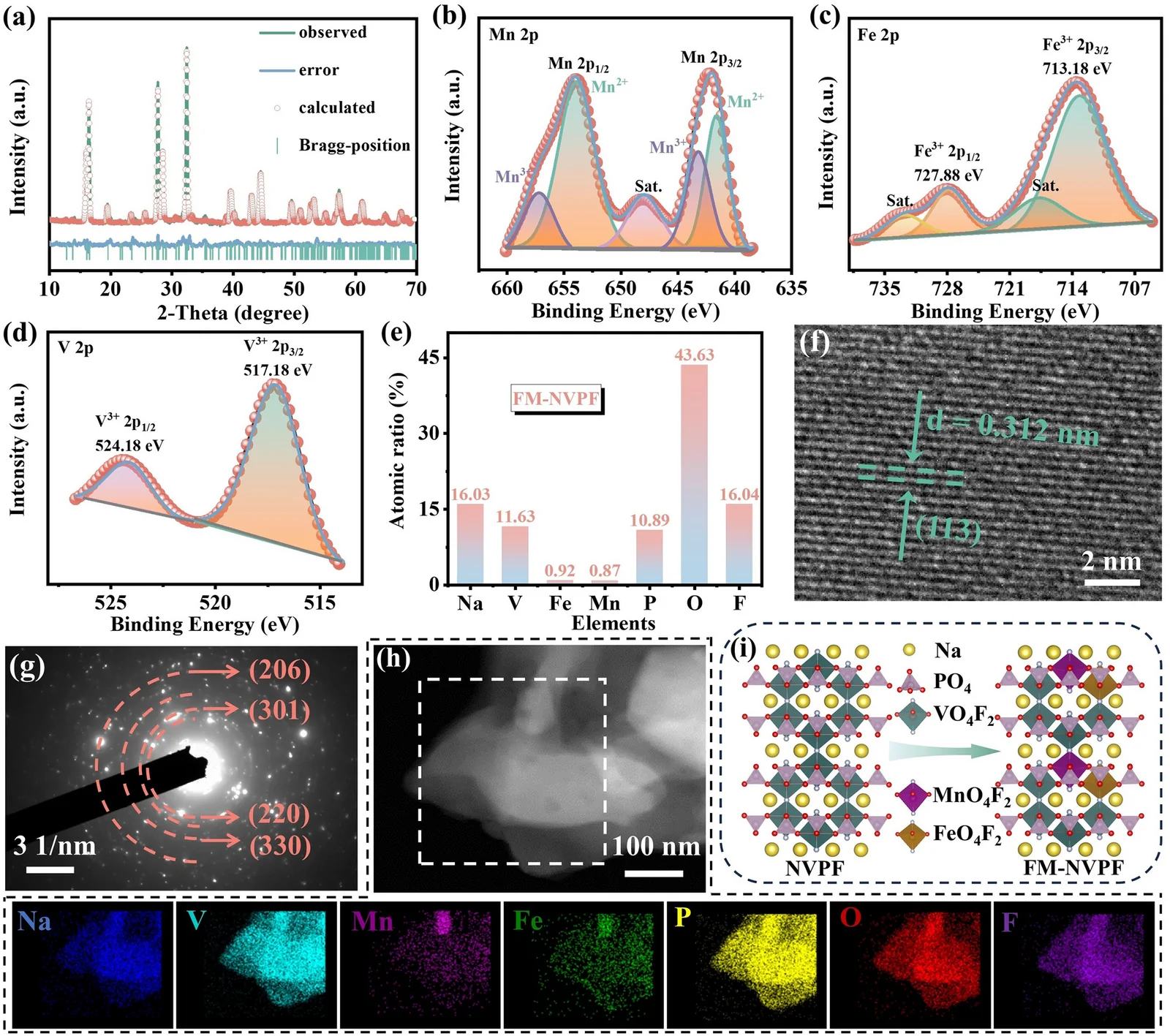
**a** Rietveld refinement profile of FM-NVPF. High-resolution XPS spectra: **b** Mn 2*p*, **c** Fe 2*p*, **d** V 2*p* spectra and **e** atomic ratio form ICP results of FM-NVPF. **f** HRTEM image and **g** Diffraction pattern of FM-NVPF. **h** STEM images and corresponding element mapping of Na, V, Mn, Fe, P, O, F for FM-NVPF. **i** Schematic illustration of the crystal structures changing from NVPF to FM-NVPF

The effect of doping concentration on the purity of NVPF phase is explored. In the XRD spectrum of Fig. S1a, with the increase in Fe and Mn doping levels, the peak of NVP impurity phase gradually weakened and disappeared. The GCD plots in Fig. S1b also verifies this point. As the concentration increases, the discharge platform at the low voltage gradually disappears, and when the concentration reaches 1.5 mmol, the capacity also reaches the maximum value. When the concentration continues to increase to 2 mmol, the capacity decays, which should be due to the fact that the non-reactive doping elements replaced too much V, resulting in a decrease in the redox reaction. Therefore, the synthesized NVPF at a concentration of 1.5 mmol (FM-NVPF) was selected as the final research object. The chemical structures and phase purity of undoped NVPF, Fe-doped NVPF (F-NVPF), Mn-doped NVPF (M-NVPF), and FM-NVPF were further compared by XRD characterization to illustrate the synergistic effect of bimetallic doping (Fig. S2a). It shows that only FM-NVPF indicates the pristine NVPF phase (PDF#89–8485) in the space group of *P42/mnm*, whereas the other samples display a small amount of NVP impurities, similar to prior reports [26, 27]. Rietveld refinement of XRD patterns yields lattice parameters for the FM-NVPF material (Fig. 1a) with I_c_ = 10.732 Å and I_a_ = I_b_ = 9.083 Å, *α* = *β* = *γ* = 90° (Table S1), and the atomic accupations are tabulated in Table S2. Its quality factor *R*_wp_* is 0.045, which is less than 0.1, indicating a good fit [28].

The chemical compositions were examined by X-ray photoelectron spectroscopy (XPS) analysis. The general XPS spectra in Fig. S2b reveal consistent elemental signals of Na, F, O, V, C, and P across all four samples. The existence of C signal is due to the fact that in the sol–gel synthesis process, oxalic acid was introduced as a complexing agent and in-situ carbon source. Upon thermal treatment under an inert atmosphere, oxalic acid undergoes pyrolytic decomposition, generating CO, CO₂, and residual carbon species. These carbonaceous fragments deposit uniformly on the surface of the precursor particles, forming a thin and conformal carbon coating. The high-resolution Mn 2*p* spectrum for FM-NVPF in Fig. 1b can be deconvoluted into Mn 2*p*_1/2_ at 654.08 eV, Mn 2*p*_3/2_ at 642.08 eV, and the characteristic satellite peak of Mn^2+^ at 647.98 eV. The two dominant peaks are assigned to Mn^2+^ (641.58 and 653.88 eV) and Mn^3+^ (643.18 and 657.18 eV) [29, 30]. Figure 1c shows the Fe 2*p*_3/2_ peak at 713.18 eV and the Fe 2*p*_1/2_ peak at 727.88 eV, referring to Fe^3+^ [31, 32]. The high-resolution spectrum of V 2*p* (Fig. 1d) shows two typical characteristic peaks of V 2*p*_3/2_ at 517.18 eV and V 2*p*_1/2_ at 524.18 eV for V^3+^ [33, 34]. The contents of Fe and Mn dopants are further quantified by using inductively coupled plasma mass spectroscopy (ICP-MS). Figure 1e shows the ICP-determined chemical formula of Na_3_V_1.73_Fe_0.14_Mn_0.13_(PO_4_)_2_F_3_ with a total doping level of 13.5 at%.

The electron energy loss spectroscopy (EELS) spectrum in Fig. S3 confirms the presence of Fe-L_3_ and Mn-L_3_ signals in the FM-NVPF sample [35]. SEM images in Fig. S4 show pristine NVPF composed of primary nanoparticles (570 nm), while F-NVPF and M-NVPF consist of larger interconnected particles (850 nm). Interestingly, FM-NVPF shows integrated microparticles composed of numerous pores, possibly due to the optimized pH conditions. BET analyses corroborate the enhanced porosity for FM-NVPF materials (Fig. S5), which can facilitate electrolyte penetration and Na ion diffusion. In Fig. S6, the TEM image reveals a uniform amorphous carbon coating layer of approximately 3 nm on the edge of the FM-NVPF. This in-situ derived carbon layer not only enhances its electrical conductivity but also improves structural integrity by buffering volume changes during cycling. The high-resolution TEM image in Fig. 1f exhibits a clear crystal lattice distance of 0.312 nm corresponding to the (113) plane of the tetragonal phase FM-NVPF, in agreement with the selected area electron diffraction (SAED) pattern in Fig. 1g [36]. HAADF-STEM image (Fig. 1h) and EDS mapping confirm the uniform elemental distributions of Na, V, Mn, Fe, P, O, and F in the porous FM-NVPF particles. Figure 1i illustrates the crystal structure models of the FM-NVPF cathode with Fe and Mn at adjacent sites, where the PO_4_ tetrahedron and MO_4_F_2_ octahedron are connected at vertex angle to form a 3D framework for Na ion diffusion.

### The Electrochemical Performance

**Fig. 2:**
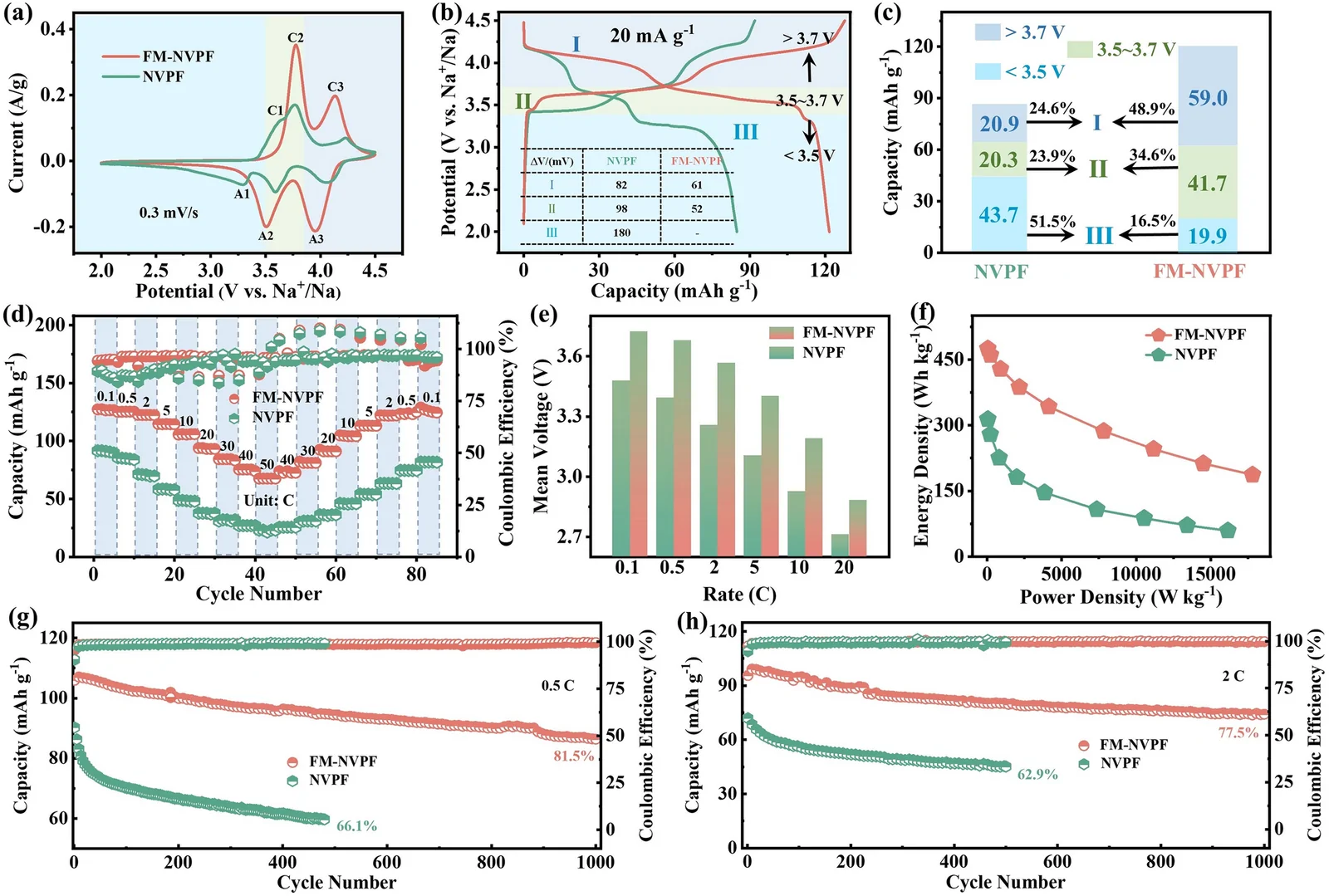
**a** CV curves and **b** discharge/charge voltage profiles for pristine NVPF and FM-NVPF (the inset table shows the overpotentials). **c** Capacity contribution ratios in three voltage intervals. **d** Cyclic capacities at increasing current rates from 0.1 C to 50 C and reverse. **e** Average operation voltage *V*_m_ at different current rates. **f** Energy and power density Ragone plots. **g, h** Cycling capacities

The electrochemical characterizations are conducted to illustrate the impact of Fe and Mn doping on the Na ion storage behaviors in NVPF cathodes. Figure 2a shows the CV curves of pristine NVPF with three redox pairs at around 3.61/3.29 V, 3.77/3.59 V, and 4.23/4.07 V, referring to C_1_ (cathodic)/A_1_ (anodic), C_2_/A_2_, and C_3_/A_3_, respectively. The C_1_/A_1_ redox pair at 3.61/3.29 V almost disappeared for FM-NVPF, which displayed enhanced redox peaks at the high voltage region (C_2_/A_2_ at 3.78/3.51 V and C_3_/A_3_ at 4.13/3.95 V). The above results are basically consistent with the previously reported literature, where the charge storage process is shown in the following formula [37, 38]:

$$Na_{3} V_{2} \left( {PO_{4} } \right)_{3} \to NaV_{2} \left( {PO_{4} } \right)_{3} + 2Na^{ + } + 2e^{ - } \;\;\;\;\;\;\;\;\;\left( {{\mathrm{C}}_{{1}} /{\mathrm{A}}_{{1}} } \right)$$

$$Na_{3} V_{2} \left( {PO_{4} } \right)_{2} F_{3} \to Na_{2} V_{2} \left( {PO_{4} } \right)_{2} F_{3} + Na_{\left( 3 \right)}^{ + } + e^{ - } \;\;\;\;\;\;\;\;\left( {{\mathrm{C}}_{2} /{\mathrm{A}}_{2} } \right)$$

$$Na_{2} V_{2} \left( {PO_{4} } \right)_{2} F_{3} \to NaV_{2} \left( {PO_{4} } \right)_{2} F_{3} + Na_{\left( 2 \right)}^{ + } + e^{ - } \;\;\;\;\;\left( {{\mathrm{C}}_{{3}} /{\mathrm{A}}_{{3}} } \right)$$

The C1/A1 peak at low voltage is attributed to the presence of NVP impurity, while the C2/A2 and C3/A3 peaks at high voltage correspond to the Na^+^ de/intercalation at the two partially-occupied sites of Na_(3)_ and Na_(2)_ sites, respectively.

Figure 2b shows that pristine NVPF exhibited a long discharge plateau at around 3.4 V, whereas FM-NVPF presented no plateau at such a low potential, but instead displayed two plateaus at 3.6 and 4.1 V. The inset table shows the polarization voltage of the two obtained from the d*Q*/d*V* curves in Fig. S7, where FM-NVPF has a smaller value, indicating its faster reaction kinetics and more efficient charge transport. Figure 2c summarizes the capacity contributions in different voltage ranges. Only 19.9% of the FM-NVPF’s Na ion storage capacities occur below 3.5 V, compared to 51.5% for pristine NVPF. In the high voltage range of above 4.0 V, the capacity contribution of FM-NVPF reaches 48.9%, which is much higher than that for pristine NVPF. Overall, the FM-NVPF cathode effectively eliminates the low voltage redox region associated with NVP impurities. The GCD plots in Fig. S8 further intuitively demonstrate the improvement of the charge storage performance by the synergistic effect of dual doping.

The rate performance of FM-NVPF cathode is further investigated under stepwise increasing current rates from 0.1 to 50 C (1 C = 128 mA g^−1^), which exhibited capacities of 126.57, 125.26, 122.32, 114.45, 105.88, 93.14, 83.19, 73.95, and 67.55 mAh g^−1^ (Fig. 2d), respectively. These values are much larger than the corresponding capacities of pristine NVPF cathodes (*i.e.,* 91.62 mAh g^−1^ at 0.1 C, 23.11 mAh g^−1^ at 50 C), indicating the remarkable reaction kinetics for FM-NVPF cathodes. As the current densities gradually return to 0.1 C, the reversible capacity for FM-NVPF recovered to its initial value, indicating excellent electrochemical reversibility. Figure 2e shows the mean voltages of 3.72 V at 0.1 C and 2.88 V at 20 C for FM-NVPF cathode, which are 0.24 and 0.17 V higher than the corresponding values for pristine NVPF. The smaller polarization and higher redox voltages are beneficial to the energy densities of FM-NVPF cathodes. Figure 2f summarizes the energy and power densities of FM-NVPF and NVPF cathodes. FM-NVPF demonstrates energy densities in the range of 474 to 187 Wh kg^−1^ at 44 to 17,807 W kg^−1^, which is much larger than these of pristine NVPF and other polyanionic cathode materials in literature [11, 26, 31, 39].

Long-cycle stability of FM-NVPF is an important indicator of their practical application. Figure 2g shows that the FM-NVPF delivers a capacity retention of 81.5% after 1000 cycles at 0.5 C, but the pristine NVPF decayed seriously with a capacity retention of 66.1% of its initial value after 500 cycles. The FM-NVPF cathodes also demonstrate exceptional cyclic stability at a high current density of 2 C with Coulombic efficiencies close to 100% (Fig. 2h). The structural integrity of FM-NVPF is further explored by extended cycling at higher current densities. As shown in Fig. S9, the FM-NVPF can be cycled for 1000 cycles at 4 and 10 C with capacity retention rates of 72.1% and 83.6%, respectively. Table 1 illustrates a comparative analysis of the electrochemical properties of NVPF-based electrode materials, which clearly illustrates the superior long-term cycling performance and high-rate capability of our FM-NVPF cathode [11, 13, 15, 26, 29, 33, 34, 36, 40, 41].

**Table 1:** Comparison of electrochemical performance for NVPF-based electrodes

NVPF-based electrode	Voltage (V versus Na^+^/Na)	Specific capacity (mAh g^−1^)	Rate capacity (mAh g^−1^)	Stability at a low rate	Stability at a high rate	References
NVM_2_PF@C	2.5–4.3	116.2 at 0.2 C	42.5 at 5 C	80.2% (100 cycles at 0.2 C)	67.7% (400 cycles at 1 C)	[29]
c-NVPF@NC	2.0–4.5	121.7 at 0.2 C	54.7 at 20 C	82.0% (500 cycles at 5 C)	77.6% (1000 cycles at 10 C)	[11]
K0.10-NVPF	2.5–4.3	120.8 at 0.1 C	66.0 at 30 C	97.5% (500 cycles at 1 C)	89.8% (500 cycles at 5 C)	[40]
NVPF-H@cPAN	2.0–4.5	116.2 at 0.2 C	58.3 at 20 C	98.1% (50 cycles 0.2 C)	85.0% (2000 cycles 5 C)	[33]
NVPF@3Dc	2.5–4.3	131.5 at 0.2 C	65.7 at 10 C	92.7% (150 cycles at 0.2 C)	63.7% (2500 cycles at 5 C)	[36]
NVPF/rGO&C	2.3–4.5	119.2 at 0.5 C	77.5 at 30 C	98.5% (200 cycles at 10 C)	81.9% (1000 cycles at 30 C)	[34]
NV_0.97_Fe_0.03_PF/C	2.5–4.3	126.7 at 0.1 C	85.0 at 10 C	97.1% (100 cycles at 0.2 C)	87.8% (300 cycles at 1 C)	[15]
NVMPF	2.5–4.6	108.5 at 0.1 C	74.0 at 10 C	95% (50 cycles at 0.5 C)	–	[41]
NVPF	2.0–4.3	119.9 at 1 C	89.0 at 30 C	81.9% (500 cycles at 1 C)	81.3% (1700 cycles at 10 C)	[26]
HE-NVPF	2.0–4.5	118.5 at 0.1 C	71.4 at 50 C	90.2% (400 cycles at 0.5 C)	80.4% (2000 cycles at 20 C)	[13]
FM-NVPF	2.0–4.5	126.6 at 0.1 C	67.6 at 50 C	81.5% (1000 cycles at 0.5 C)	83.6% (1000 cycles at 10 C)	This work

### Charge Storage Mechanism and Kinetics Analysis

To elucidate the mechanisms underlying the impressive electrochemical performance of FM-NVPF, we performed a series of electrochemical tests to study the Na ion diffusion kinetics. Figure S10a presents the EIS curves for the FM-NVPF and pristine NVPF cathodes. The FM-NVPF electrode presented a smaller semicircle at the mid-frequency region, indicative of reduced charge transfer resistance (*R*_ct_) [42]. The slop of the Warburg tail at the low-frequency region reflects the ion diffusion kinetics [19], with FM-NVPF displaying a steeper slop than pristine NVPF, suggesting enhanced ion diffusion kinetics. In Fig. S10b, by fitting the Warburg region, the *D* value of FM-NVPF was calculated as 1.31 × 10^–13^ cm^2^ s^−1^, which is significantly higher than the 6.56 × 10^–15^ cm^2^ s^−1^ for pristine NVPF. Detailed parameters for calculating the above *D* values are summarized in Table S4.

**Fig. 3:**
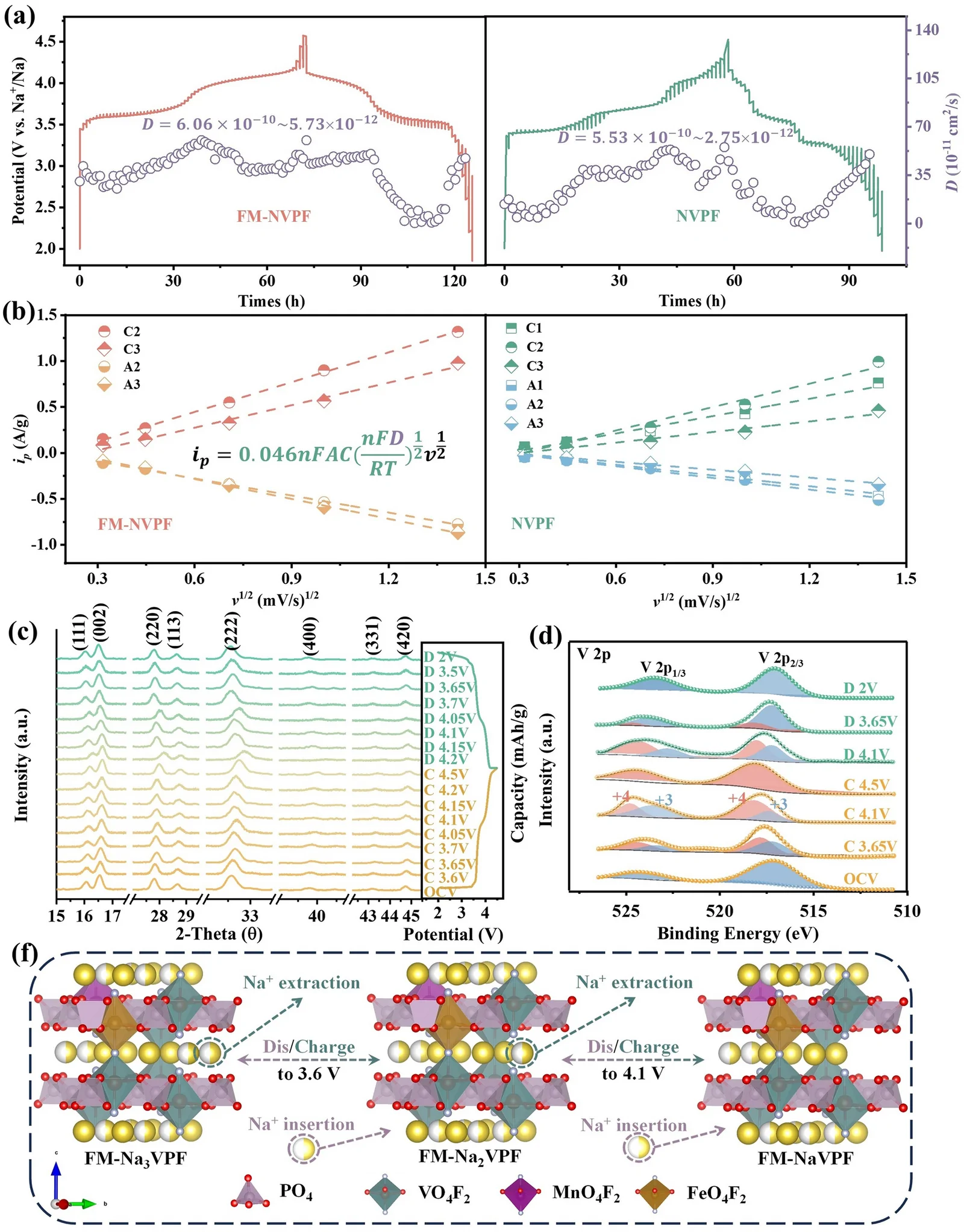
**a** Na ion diffusion coefficients during charging and discharging calculated by the GITT method. **b** *i*_p_ as a function of *v*^1/2^. **c** Ex-situ XRD patterns and **d** V 2*p* XPS spectra of FM-NVPF at different discharging/charging stages. **f** Schematic illustration of the Na^+^ insertion and extraction mechanisms in FM-NVPF

Additional galvanostatic intermittent titration technique (GITT) and CV tests at different scanning rates also corroborate these findings. Figure 3a shows the GITT plots and the corresponding diffusion coefficient. *D* values range from 5.73 × 10^–12^ to 6.06 × 10^–10^ cm^2^ s^−1^ for FM-NVPF during (de)sodiation, which is larger than those of pristine NVPF. The superior Na ion diffusion coefficient can also be supported by the CV test in Fig. 3b. Figure S11 shows the CV curves at different scan rates ranging from 0.1 to 2 mV s^−1^ for pristine NVPF and FM-NVPF cathodes. The Na ion storage mechanisms can be qualitatively analyzed using the equation of *i*_*p*_ = *av*^*b*^ [43], where* i*_*p*_ is the peak current density, *v* is the scan rate, *b* = 0.5 refers to diffusion-controlled reaction, and b = 1 for capacitive limited reaction. The *b* value can be determined by the log(*i*_*p*_) *versus* log(*v*) plots [44]. As shown in Fig. S12, the *b*-values of the A2/C2 and A3/C3 redox peaks for FM-NVPF are 0.73/0.67 and 0.83/0.78, respectively, indicating that the charge storage behavior is predominantly diffusion controlled with a partial capacitive contribution. In comparison, the corresponding *b*-values for NVPF and NVP exhibit a slight increase, suggesting an enhanced contribution from capacitive processes.

The Na ion storage mechanisms in FM-NVPF are investigated by *ex-situ* XRD and XPS characterizations. Figure 3c shows that during charging, the extraction of Na ions and valence state change of the vanadium redox center led to a decrease in the basal plane as shown by the right shift of (002) and (222) planes. During the subsequent discharge process, the diffraction peaks return to their initial positions. No apparent discrepancy is observed for the pristine and fully discharged peaks, indicating highly reversible sodiation/desodiation reactions. However, for NVPF (Fig. S13), as charging and discharging process reaches a high potential, the peaks corresponding to the (111), (002), and (331), (420) crystal planes show obvious peak disappearance and the formation of new peaks, indicating the existence of phase change, which will affect the structural stability of the material. Figure 3d presents the V 2*p* XPS spectra for FM-NVPF after discharging and charging. FM-NVPF electrodes existed in a V^3+^ valent state. When charging to 3.65 V, corresponding to FM-N_x_VPF (*x* = 0.5), a V^4+^ peak appears along with the increase in the average valence state of V. With further charging to 4.5 V, the completed deintercalation of two Na^+^ resulted in the full oxidation of V^3+^ into V^4+^. With the insertion of Na ions during discharging, the valence state of V^3+^ can be completely recovered.

Whether Fe and Mn elements participate in the reaction during the charging/discharging process was also further explored. The peak of Fe 2*p*_3/2_ spectrum shown in Fig. S14a that does not change during the charge/discharge process, indicating that Fe does not participate in the electrode reaction. The Mn 2*p*_3/2_ spectrum (Fig. S14b) shows that its peak moves toward a higher binding energy as the charge progresses, and returns to a low binding energy state during the discharge process, indicating a reversible valence change (Mn^2+^/Mn^3+^) during the electrode reaction. At this time, it is easy to induce Jahn–Teller distortion related to Mn^3^⁺ (d^4^), resulting in local structural asymmetry and damage to structural stability. However, Fe/Mn co-doping can effectively suppress this phenomenon. Fe^3^⁺ is a half-full shell of *d*^5^, Jahn–Teller is inactive, and the concentration of JT active centers in the material can be diluted. In addition, co-doping can also promote electron delocalization and lattice relaxation, which is conducive to the stability of the overall crystal structure and the smoothness of the ion diffusion path.

The in-situ EIS spectra of FM-NVPF reveal a non-monotonic evolution during the charge–discharge process (Fig. S15a). During the initial charge, the charge transfer resistance (*R*_ct_) decreases due to the gradual activation of Na⁺ diffusion pathways as partial desodiation opens up the NASICON framework. As charging proceeds beyond 3.8 V, *R*_ct_ increases significantly, likely due to lattice contraction, increased polarization, and surface reactions at higher voltages. Concurrently, the low-frequency Warburg slope becomes steeper, suggesting accelerated ion diffusion after the removal of Na⁺ from blocked sites. Conversely, during discharge, a gradual increase in *R*_ct_ is observed, possibly due to structural swelling, uneven Na⁺ reinsertion, or surface side reactions. The Warburg slope correspondingly flattens, indicating sluggish Na⁺ diffusion. As the discharge is completely completed, the curve basically returns to the initial state, indicating its good electrochemical reversibility. These dynamic impedance features reflect the complex interplay between lattice structure, charge transport, and interfacial stability during electrochemical cycling. In Fig. S15b, the variation trend of NVPF is basically consistent with that of FM-NVPF, but its *R*_ct_ value is larger throughout the whole process, and the slope of the straight line in the low-frequency region is lower, indicating its lower charge transfer and ion diffusion efficiency. Figure S16 shows the GCD plots corresponding to the in-situ EIS spectra. Figure 3f illustrates the charge storage mechanism within the FM-NVPF crystal structure during charge/discharge, highlighting the highly reversible Na ion insertion and extraction processes.

### Electronic Structure Regulation Analysis

**Fig. 4:**
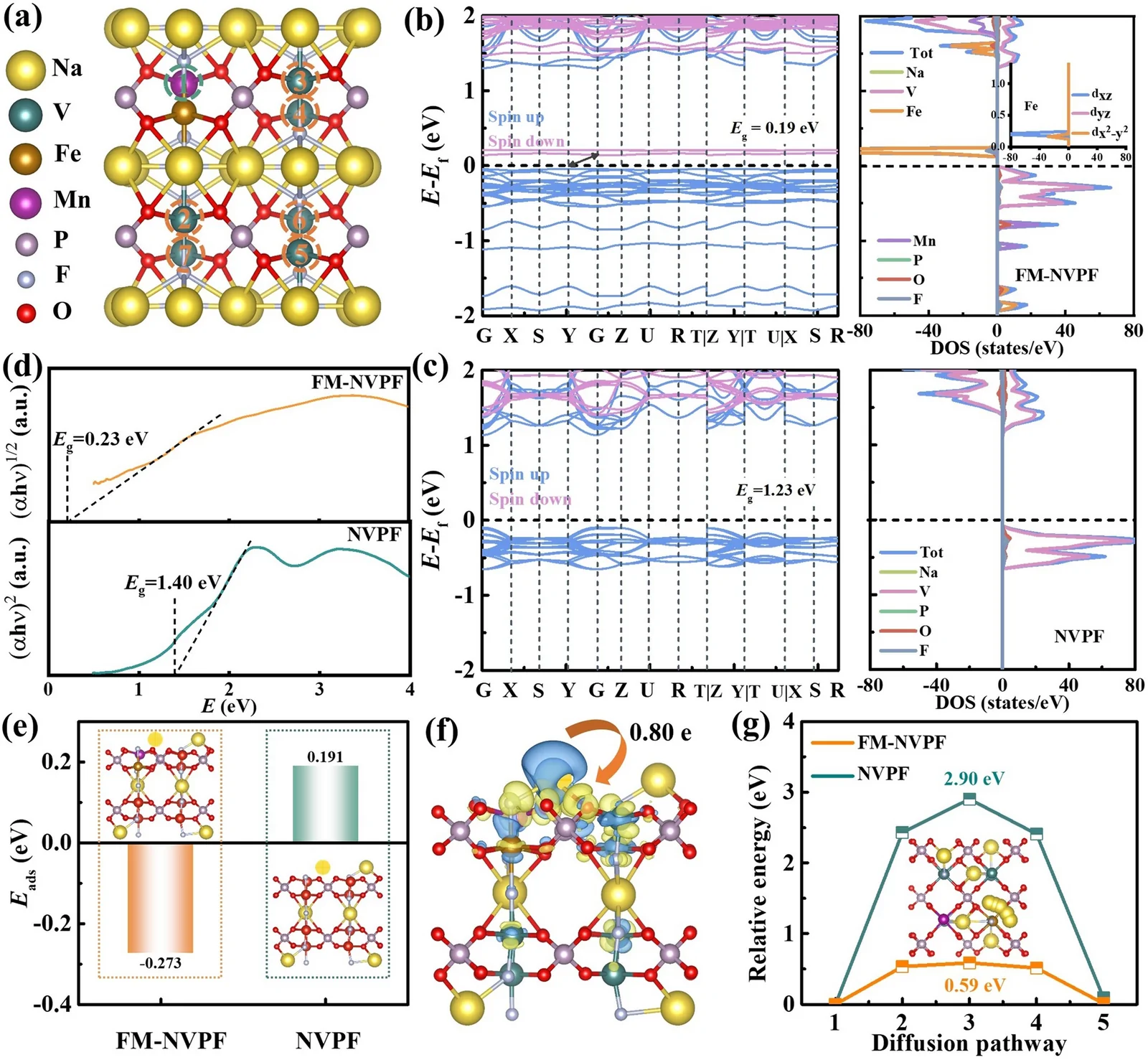
**a** Distribution of Fe and Mn doping sites on NVPF. Electronic band structure and density of states of **b** FM-NVPF and **c** NVPF. **d** Band gap energy estimations based on the Kubelka–Munk function. **e** Adsorption energies (*E*_ads_) of Na. **f** Bader charge density difference for Na adsorption on FM-NVPF. The blue and yellow regions represent electron depletion and accumulation, respectively, with the isosurface value set at 0.002 e⋅Å⁻^3^. **g** Diffusion energy profiles of Na with the inset displaying the top view of the diffusion pathway on FM-NVPF

DFT theoretical calculations are used to investigate the distribution of Fe and Mn dopants, the electronic properties of FM-NVPF electrodes, and the dynamic effects of Na diffusion on the FM-NVPF surface. ICP-MS results indicate a doping ratio of Fe: Mn ≈ 1:1 in Na_3_V_1.73_Fe_0.14_Mn_0.13_(PO_4_)_2_F_3_. Figure 4a illustrates the possible Mn doping sites, and Table S5 presents the total energy of FM-NVPF with Mn at various sites. The results indicate that the closest proximity of Mn to Fe results in the lowest energy structure for the system. The high electronic conductivity of electrode materials is critical for maintaining the rate performance and cycling stability. To investigate the influence of co-doping on the intrinsic electronic properties of the material, the energy band structures and electron density of states (DOS) of Fe/Mn co-doped NVPF (Fig. 4b), Fe-doped NVPF (F-NVPF, Fig. S17a), Mn-doped NVPF (M-NVPF, Fig. S17b), and pristine NVPF (Fig. 4c) were calculated. The band calculations reveal that the direct bandgap of pristine NVPF transfers to an indirect bandgap in FM-NVPF, as the conduction band minimum (CBM) and the valence band maximum (VBM) are located at different positions in momentum space (k-points). An indirect band gap typically exhibits higher electron mobility, lower recombination rates, longer carrier lifetimes, and exceptional chemical stability in electrochemical environments [45–47]. For single-doped F-NVPF and M-NVPF, they exhibit characteristics of an indirect bandgap and a direct bandgap, respectively, suggesting that the influence of Fe/Mn co-doping on the bandgap is primarily dominated by the Fe element.

Band structure analysis reveals a bandgap reduction from 1.23 eV (pristine NVPF) to 0.19 eV (FM-NVPF), with F-NVPF and M-NVPF exhibiting bandgaps of 0.10 eV and 1.40 eV, respectively. DOS analysis reveals that Fe/Mn co-doping induces 3*d* orbital of Fe with spin-down states near the Fermi level, forming distinct defect levels (isolated sharp peaks) that significantly reduce the bandgap [48]. In contrast, Fe doping leads to hybridization between Fe-3*d* and V-3*d* orbitals near the Fermi level, resulting in continuous band formation and bandgap reduction. While Mn doping exhibits negligible bandgap modulation. Furthermore, the inset of Fig. 4b displays the projected density of states (pDOS), indicating that the defect-level states primarily originate from the d_xy_, d_yz_, and d_x2-y2_ orbitals of Fe (The d_xy_ and d_yz_ orbitals overlap).

The optical properties of FM-NVPF, F-NVPF, M-NVPF, and pristine NVPF were further characterized using UV–visible spectroscopy (UV–vis) in the range of 200–1700 nm based on the Kubelka–Munk function (Fig. S18a). The absorption edge of F-NVPF exhibited highest intensity in the visible light region (500–800 nm), suggesting that Fe doping effectively enhances visible light absorption. This enhancement can be attributed to the inherent modulation of electronic and band structures in the doped material [49]. The bandgap energy was calculated using the formula *αhv* = A(*hv*—*E*_g_)^n^, where *α* is the absorption coefficient, *h* is Planck’s constant, *E*_g_ is the bandgap, *v* is the frequency of light, and *n* corresponds to the characteristics of semiconductor transitions (*n* = 2 for direct bandgap materials and *n* = 1/2 for indirect bandgap materials) [50]. As shown in Figs. 4d and S18b, c, the bandgaps of FM-NVPF, F-NVPF, M-NVPF, and pristine NVPF were calculated to be 0.23, 0.15, 1.41, and 1.40 eV, respectively, exhibiting excellent agreement with our theoretical values of 0.19, 0.10, 1.40, and 1.23 eV while maintaining consistent trends. These findings demonstrate that Fe/Mn co-doping effectively creates defect-assisted transport pathways, enhancing ionic conductivity through improved charge carrier mobility. To more directly explore the improvement of material conductivity by dual-doping, four-point probe are used to test the conductivity of NVPF and FM-NVPF. As shown in Table S6, the conductivity of FM-NVPF (1.71E-3 mS cm^−1^) is higher than that of NVPF (1.10E-3 mS cm^−1^).

The adsorption energies of Na on FM-NVPF and pristine NVPF are simulated during the charge and discharge process, as shown in Fig. 4e. The positive adsorption energy (0.191 eV) of NVPF indicates that Na cannot be stably adsorbed on its surface. FM-NVPF exhibits negative adsorption energy, indicating that doping enhances the interaction between Na and the electrode surface, allowing Na to stably adsorb onto the electrode. Moreover, the moderate adsorption energy (−0.273 eV) facilitates ion desorption, thereby improving the reaction kinetics. The charge difference density distribution of adsorbed ions on the FM-NVPF electrode surface is further illustrated in Fig. 4f. The blue region represents electron depletion, while the yellow region indicates electron accumulation. It is evident that during the adsorption process, electrons are transferred from the Na atom to the electrode. Moreover, Bader charge analysis quantitatively determined the amount of electron transfer during adsorption to be 0.80e. The significant charge transfer is attributed to the interaction between the substrate surface and the electronegative oxygen atoms.

Figure 4g shows the Na ion diffusion energy barriers of FM-NVPF and NVPF, with the inset displaying the migration pathway of FM-NVPF. The Na ion diffusion pathway in pristine NVPF is in Fig. S18d. The migration energy barrier of FM-NVPF is 0.59 eV, significantly lower than that of NVPF (2.90 eV), indicating that the reduced barrier in FM-NVPF facilitates faster Na ion diffusion, thereby suppressing Na site rearrangement and the phase transition process in the low voltage region. This superior reaction kinetics and rate performance result from reduced ion migration barriers and enhanced electronic conductivity.

### Full Cell Performance

**Fig. 5:**
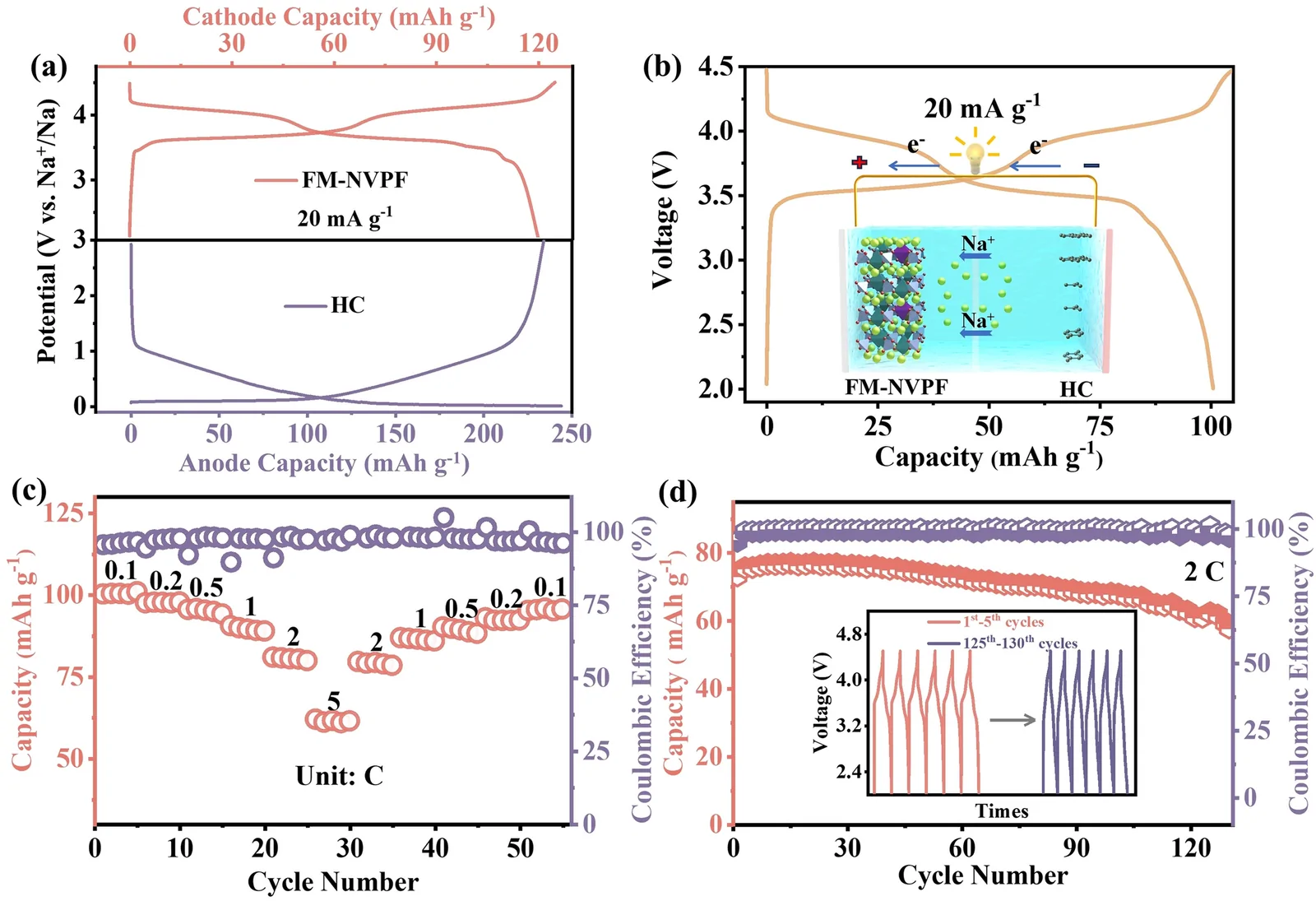
**a** Galvanostatic discharge/charge voltage profiles of the FM-NVPF cathode and HC anode in Na ion half cells. **b** Capacity–voltage profiles of FM-NVPF//HC full cell, inset shows the full cell configuration. **c** Rate capability and **d** cycling performance of the Na ion full cells

Finally, we assembled FM-NVPF//hard carbon (HC) full cells to evaluate their practical feasibility. The voltage profiles for FM-NVPF and hard carbon electrodes in Na ion half cells are plotted in Fig. 5a, which indicates high capacities and distinguished redox plateaus for both electrodes. Figure 5b shows the voltage-capacity plots of the full cells in the voltage range of 2–4.5 V at 20 mA g^−1^, which present a reversible capacity of around 100 mAh g^−1^ with high Coulombic efficiencies. Figure 5c demonstrates the full cells possess capacities of 100.9, 97.8, 94.1, 88.9, 80.3, and 61.4 mAh g^−1^ at 0.1, 0.2, 0.5, 1, 2, and 5 C, respectively, with capacities returning to their corresponding values when the current densities are stepwise decreased. The cycle stability of the full cell is illustrated in Fig. 5d with around 80% retention of the initial capacity after 130 cycles at 2 C. Under deep discharge and charge at 0.5 C, 76.3% of its initial capacity was retained after 80 cycles (Fig. S19). Compared with half-cell configurations employing excess sodium metal, the full cell exhibits relatively inferior cycling stability. This degradation is primarily attributed to two interrelated factors. First, the significant initial irreversible capacity of HC, stemming from solid electrolyte interphase (SEI) formation, consumes a substantial portion of the limited Na⁺ supplied solely by the cathode, leading to irreversible sodium loss and a depressed initial coulombic efficiency. In addition, continuous SEI growth and parasitic reactions at the low potential of HC further accelerate performance deterioration [51]. Overall, our FM-NVPF cathode materials demonstrate significant potential for practical Na ion battery applications.

## Conclusions

In summary, we developed pure-phase and co-doped FM-NVPF cathode materials for high-performance Na ion batteries through a straightforward sol-gel method. The doping ions effectively stabilize fluorine in the sol–gel precursors thus effectively suppressing the formation of low voltage NVP impurities. The FM-NVPF cathodes exhibit an exceptional capacity of 126.6 mAh g^−1^ at 0.1 C and remarkable cyclic stability with 81.5% capacity retention after 1000 cycles at 0.5 C. The significantly enhanced electrochemical performance was attributed to the ameliorated ion diffusion coefficient and electronic conductivity as illustrated in electrochemical measurements. DFT calculations revealed the regulatory mechanism of Fe and Mn doping on the intrinsic electronic structure of NVPF, which effectively improved the conductivity and reduced the energy barrier for Na ion diffusion. Ex-situ XRD and XPS analyses further demonstrate the highly reversible Na ion insertion and extraction behaviors accompanied by the reversible reduction/oxidation of the vanadium center. The practical feasibility of the FM-NVPF cathode was finally demonstrated by the stably cycling FM-NVPF//HC full cells. This study highlights the critical impact of phasic purity and heteroatom doping on the polyanionic cathode materials for the next-generation Na ion batteries.

## Supplementary Information

Below is the link to the electronic supplementary material.

Supplementary file1 (DOCX 6306 KB)
