# Supplementary material for: Iron–Manganese Dual-Doping Tailors the Electronic Structure of Na3V2(PO4)2F3 for High-Performance Sodium-Ion Batteries
Source: Nanomicro Lett. 2026 Jan 5;18:176. doi: 10.1007/s40820-025-01881-3 (PMC12765790; doi:10.1007/s40820-025-01881-3)
Supplement: Supplementary file 1 — Supplementary file1 (DOCX 6306 KB) [file 40820_2025_1881_MOESM1_ESM.docx]

Supporting Information for

**Iron-Manganese Dual-Doping Tailors the Electronic Structure of Na_3_V_2_(PO_4_)_2_F_3_ for High-Performance Sodium-Ion Batteries**

Jien Li^1, 4, #,^ *, Shuang Luo^2, #^ , Renjie Li^1^, Yingkai Hua^1^, Linlong Lyu^1^, Xiangjun Pu^1^, Jun Fan^2^, Zheng-Long Xu^1, 3,^ *

^1^ Department of Industrial and Systems Engineering, the Hong Kong Polytechnic University, Hung Hom, Hong Kong 999077, P. R. China

^2^ Department of Materials Science & Engineering, City University of Hong Kong, Hong Kong 999077, P. R. China

^3^ Research Institute for Advanced Manufacturing, the Hong Kong Polytechnic University, Hung Hom, Hong Kong 999077, P. R. China

^4^ School of Resources, Environment and Materials, Guangxi University, Nanning 530004, P. R. China

^#^Jien Li and Shuang Luo have contributed equally to this work.

*Corresponding authors. E-mail: [ljen@gxu.edu.cn](mailto:ljen@gxu.edu.cn) (Jien Li); [zhenglong.xu@polyu.edu.hk](mailto:zhenglong.xu@polyu.edu.hk) (Zheng-Long Xu)

**Supplementary Figures and Tables**


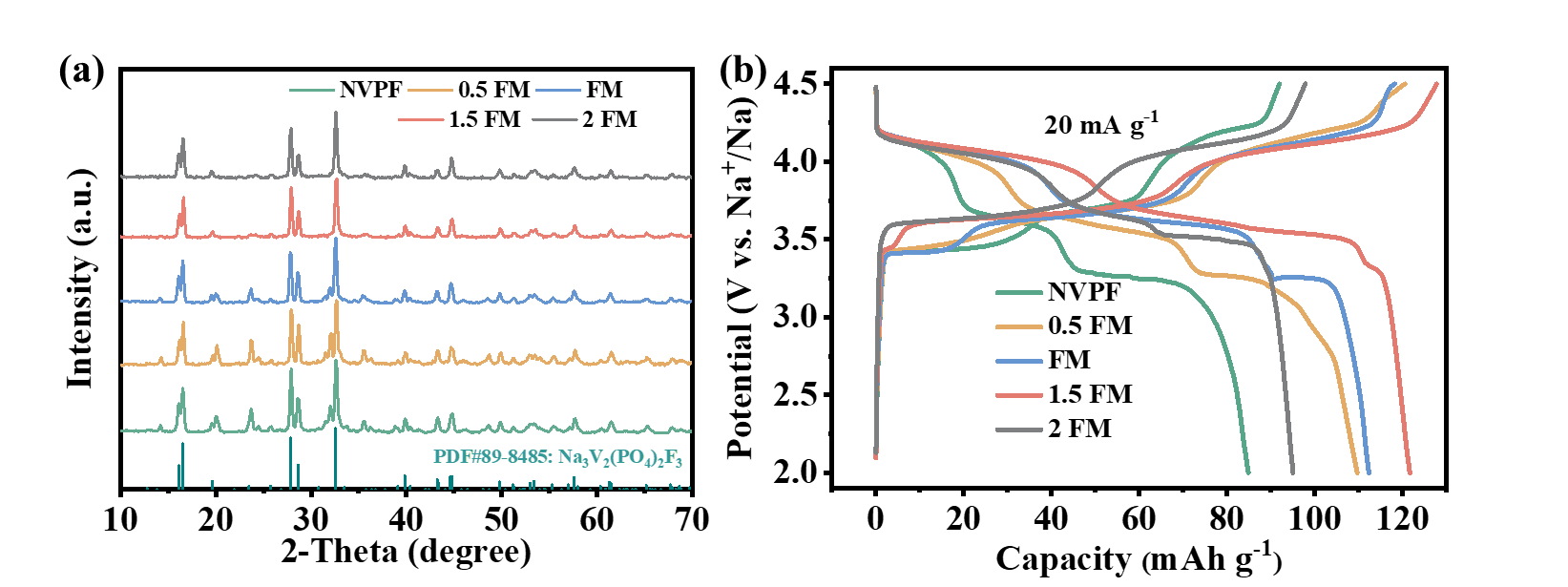


**Fig. S1** Comparison of (**a**) XRD patterns and (**b**) GCD plots for NVPF with different doping concentrations

**
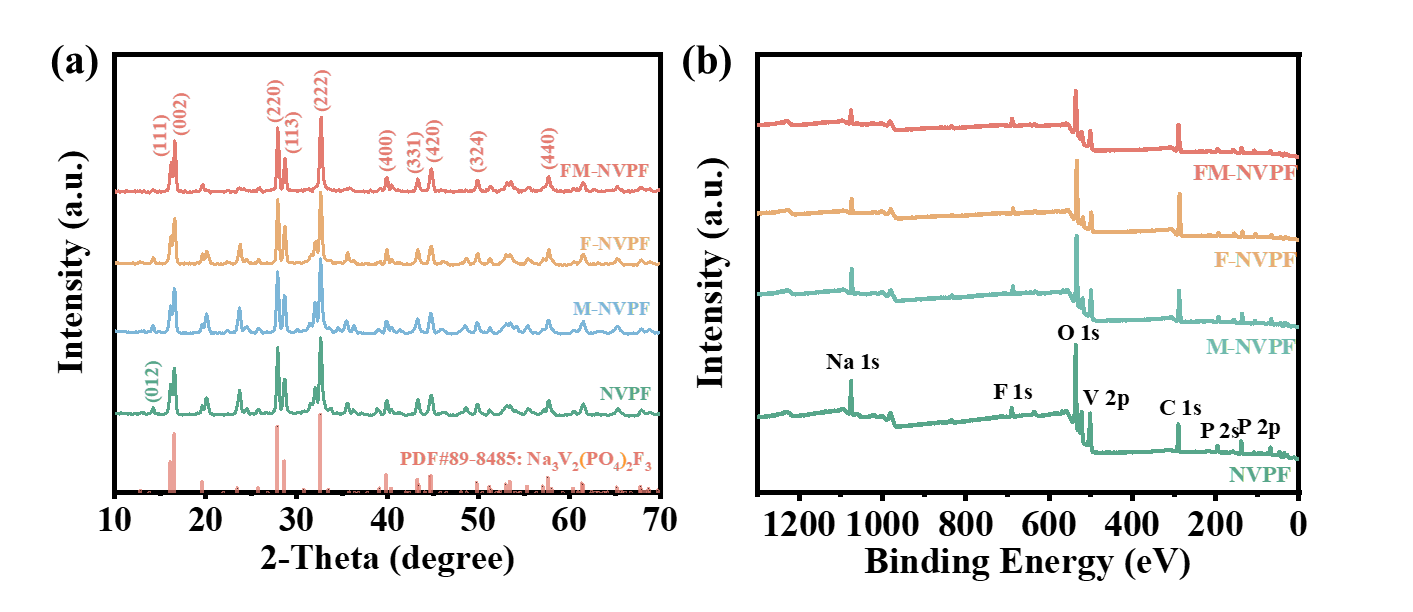
**

**Fig. S2** Comparison of (**a**) XRD patterns and (**b**) survey XPS spectra for pristine NVPF, M-NVPF, F-NVPF and FM-NVPF.

The phase purity of FM-NVPF inspired our further scrutinization of the NVPF synthesis parameters. During the sol gelling process, fluoride ions (F^-^) can volatilize under high temperatures in the form of HF, leading to the substitution of F^-^ by OH^-^ to form Na_3_V_2_(PO_4_)_2_F_3-x_(OH)_x_ precursor, which subsequently anneals into Na_3_V_2_(PO_4_)_3_ [S1]. The reactions are as follows:

${Na}_{3}V_{2}{({PO}_{4})}_{2}F_{3}+{xH}_{2}O\to{Na}_{3}V_{2}{({PO}_{4})}_{2}F_{3-x}{(OH)}_{x}+xHF$ (Reaction I)

${Na}_{3}V_{2}{({PO}_{4})}_{2}F_{3-x}{(OH)}_{x}\to{Na}_{3}V_{2}{({PO}_{4})}_{2}F_{3-y}O_{z}+{nH}_{2}O$ (Reaction II)

${Na}_{3}V_{2}{({PO}_{4})}_{2}F_{3-y}O_{z}\to{{Na}_{3}V}_{2}{({PO}_{4})}_{3}+V_{2}O_{3}+others$ (Reaction III)

Accordingly, by reducing the concentration of H⁺ ions in the solution and elevating the pH, the formation of HF can be effectively suppressed, thereby minimizing F volatilization. However, an excess of OH⁻ ions can directly substitute for F, leading to the formation of impurity phases. Therefore, it is crucial to carefully control the pH within an optimal range to prevent the formation of these phases. The addition of Fe and Mn ions acts as positive charge centers that further capture F-, thus maintaining stoichiometric ratios and suppressing the formation of impurity phases [S2-S4]. The XPS results further confirmed the increase in F content of the material after doping (Table S3).

**Fig. S3** EELS spectrum of FM-NVPF

The presence of Fe-L_3_ and Mn-L_3_ signals in the EELS spectrum further validates the successful incorporation of the bimetal dopants.


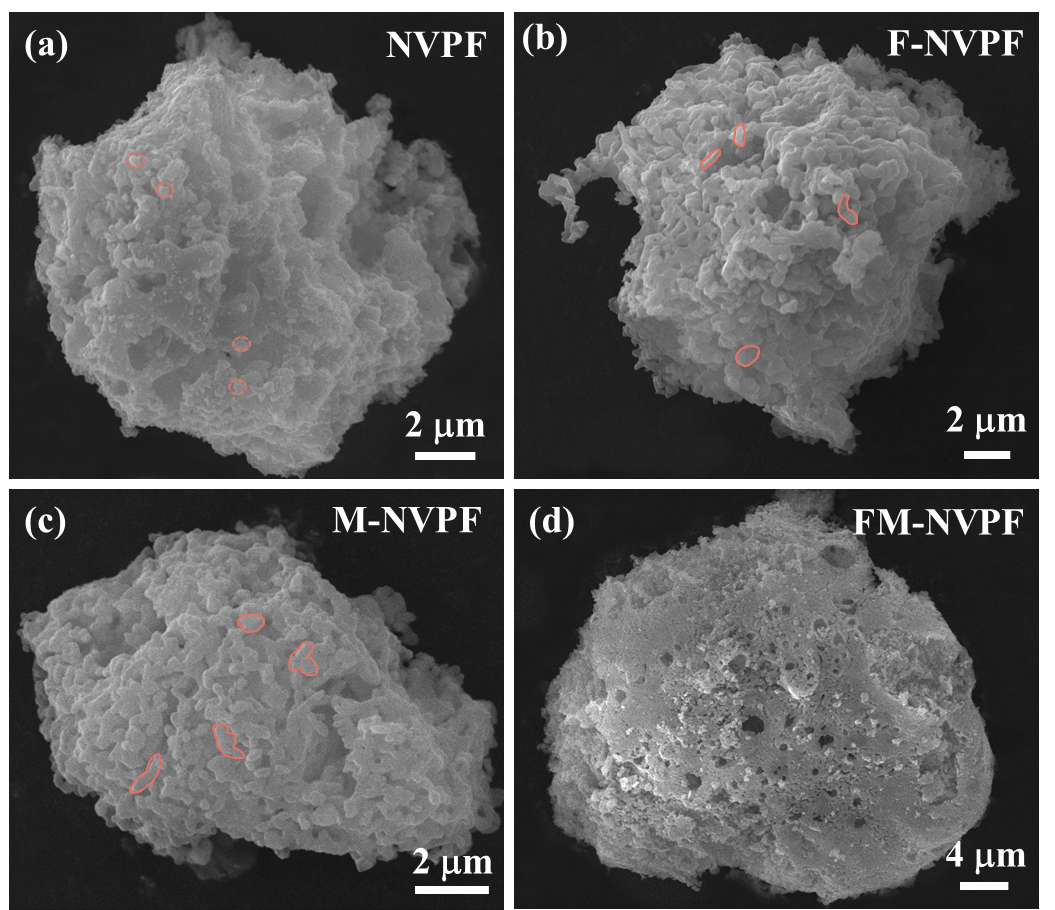


**Fig. S4** (**a-d**) Comparison of SEM images for NVPF/MVP, M-NVPF, F-NVPF and FM-NVPF

A comparison of the SEM images reveals that transition metal doping influences material nucleation, thereby modifying the morphology and microstructure. Specifically, dual doping with Fe and Mn facilitates crystal nucleation, resulting in smaller particle sizes and the formation of a multi-void structure.

**
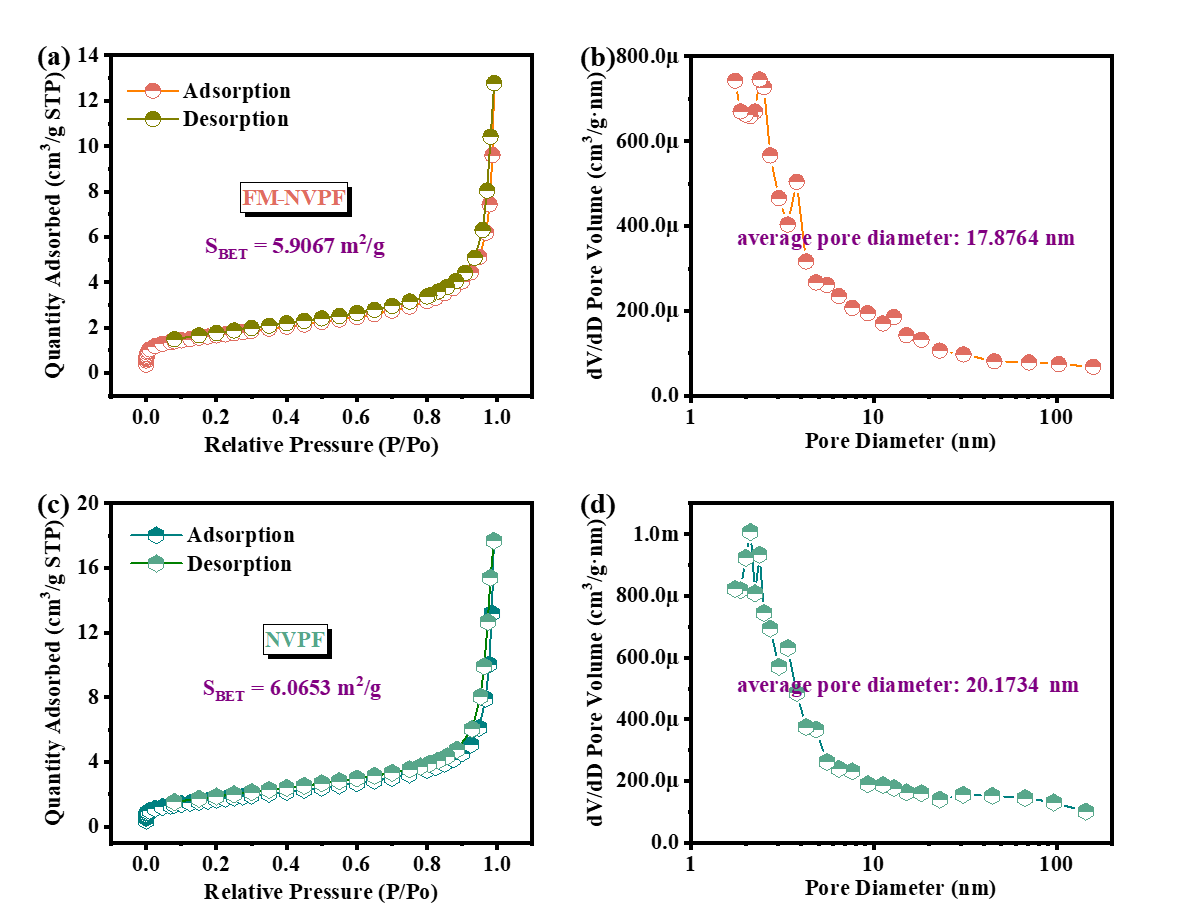
**

**Fig. S5** (**a, c**) N_2_ adsorption-desorption isotherms and (**b, d**) pore size distribution curves of the FM-NVPF and NVPF

The nitrogen desorption/adsorption plots further confirm the pore structure of the material surface. In Fig. S5a, c, both curves have an obvious hysteresis loop, indicating that they are typical mesoporous materials. The pore size distribution diagrams in Fig. S5b, d show that the main pore size distribution of both is below 10 nm, where the average pore size of FM-NVPF is 17.88 nm, while the average pore size of NVPF is 20.17 nm.


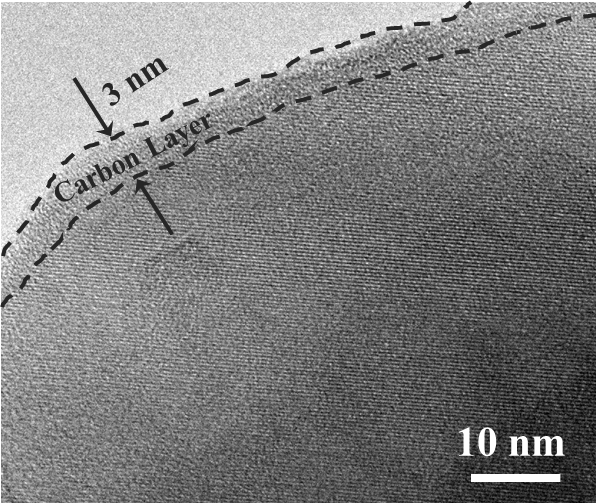


**Fig. S6** TEM image of FM-NVPF

**Fig. S7** d*Q*/d*V* curves for NVPF/NVP and FM-NVPF

**Fig. S8** Comparison of GCD plots for pristine NVPF, M-NVPF, F-NVPF and FM-NVPF

**
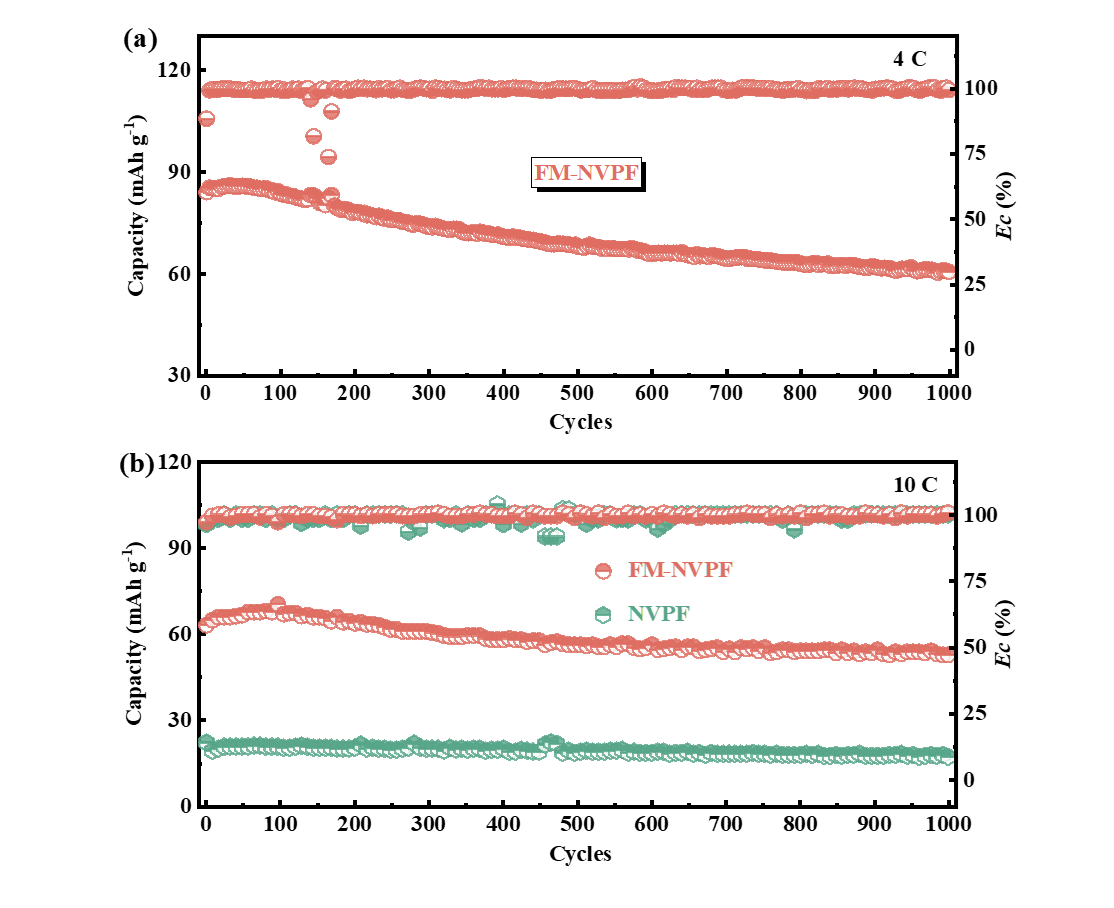
**

**Fig. S9** (**a**) The cycle performance of FM-NVPF at 4 C. (**b**) The comparison of cycle performance for FM-NVPF and NVPF at 10 C

The long-cycle stability test further confirms that FM-NVPF exhibits superior electrochemical stability at high rates.


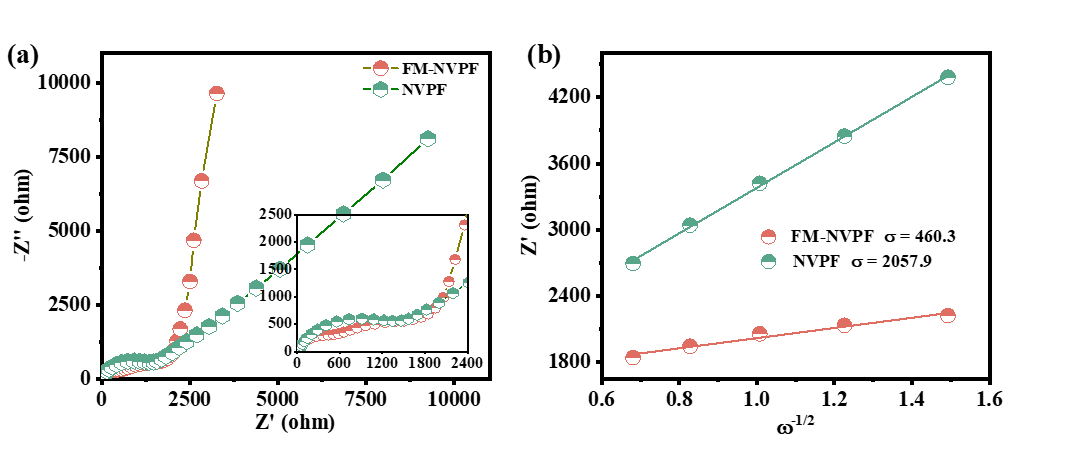


**Fig. S10** (**a**) The EIS plots and (**b**) the corresponding σ values of FM-NVPF and NVPF

According to the equation to calculate diffusion coefficient (*D*) [S5]:

| $D=\frac{R^{2}T^{2}}{2A^{2}n^{4}F^{4}C^{2}\sigma^{2}}$ | (S1) |
| --- | --- |

where *σ* is the Warburg impedance, *ω* is the angular frequency, *R* (J/mol⋅K) is the gas constant, *T* (K) is the absolute temperature, *A* (cm^2^) is the electrode area, *n* is the number of electrons transferred, *F* (C/mol) is the Faraday’s constant, *C* (mol/L) is the concentration of Na^+^.


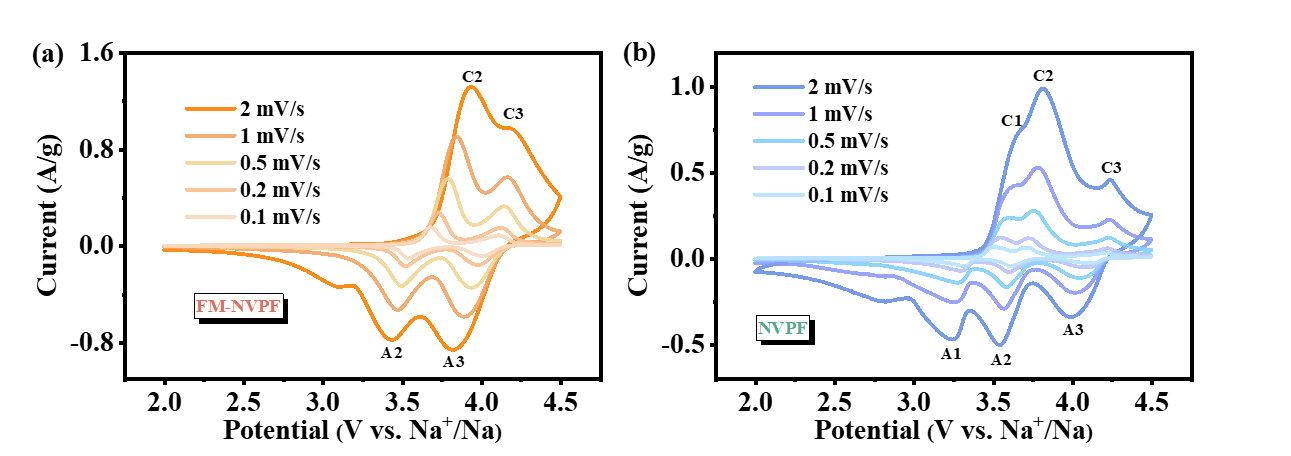


**Fig. S11** The CV curves of (**a**) FM-NVPF and (**b**) NVPF at different scan rates

The CV curves at different scan rates of NVPF have three pairs of redox peaks. However, in the curves of FM-NVPF, due to the disappearance of the NVP impurity phase, the peak at the 3.4 V low potential platform has disappeared, which is consistent with the previous GCD test results.


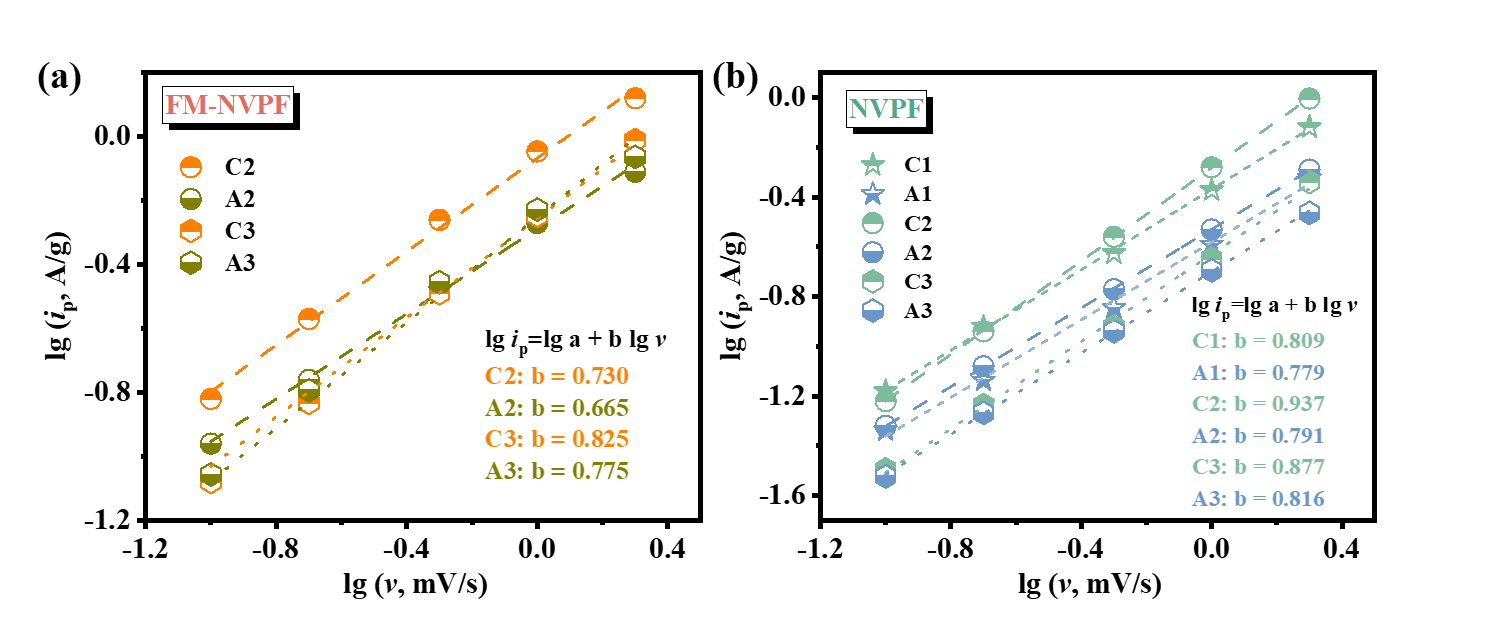


**Fig. S12** b values determined by lg (*i*_p_) against lg (*v*) of (**a**) FM-NVPF and (**b**) NVPF.


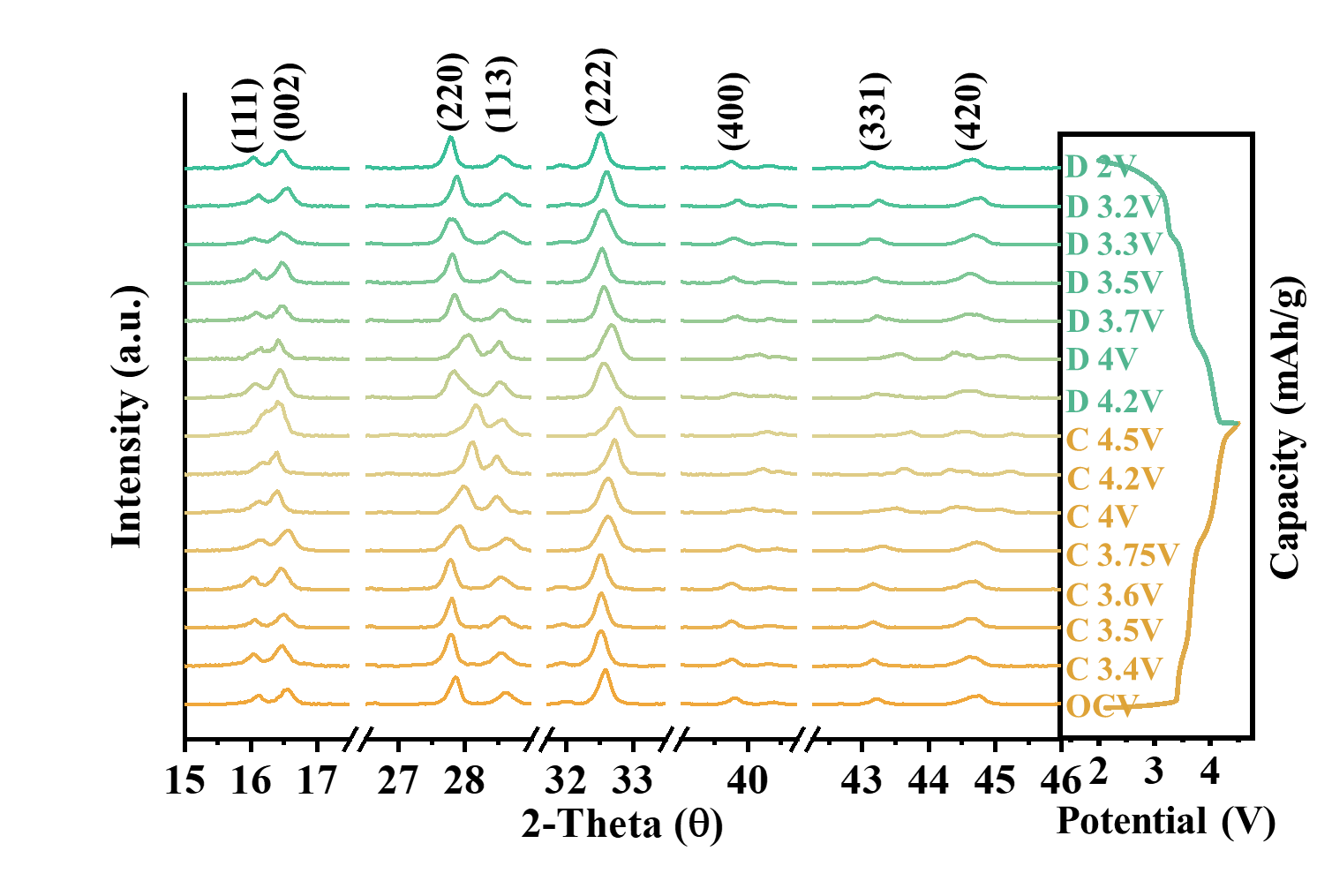


**Fig. S13** Ex-situ XRD patterns of NVPF at different discharging/charging stages


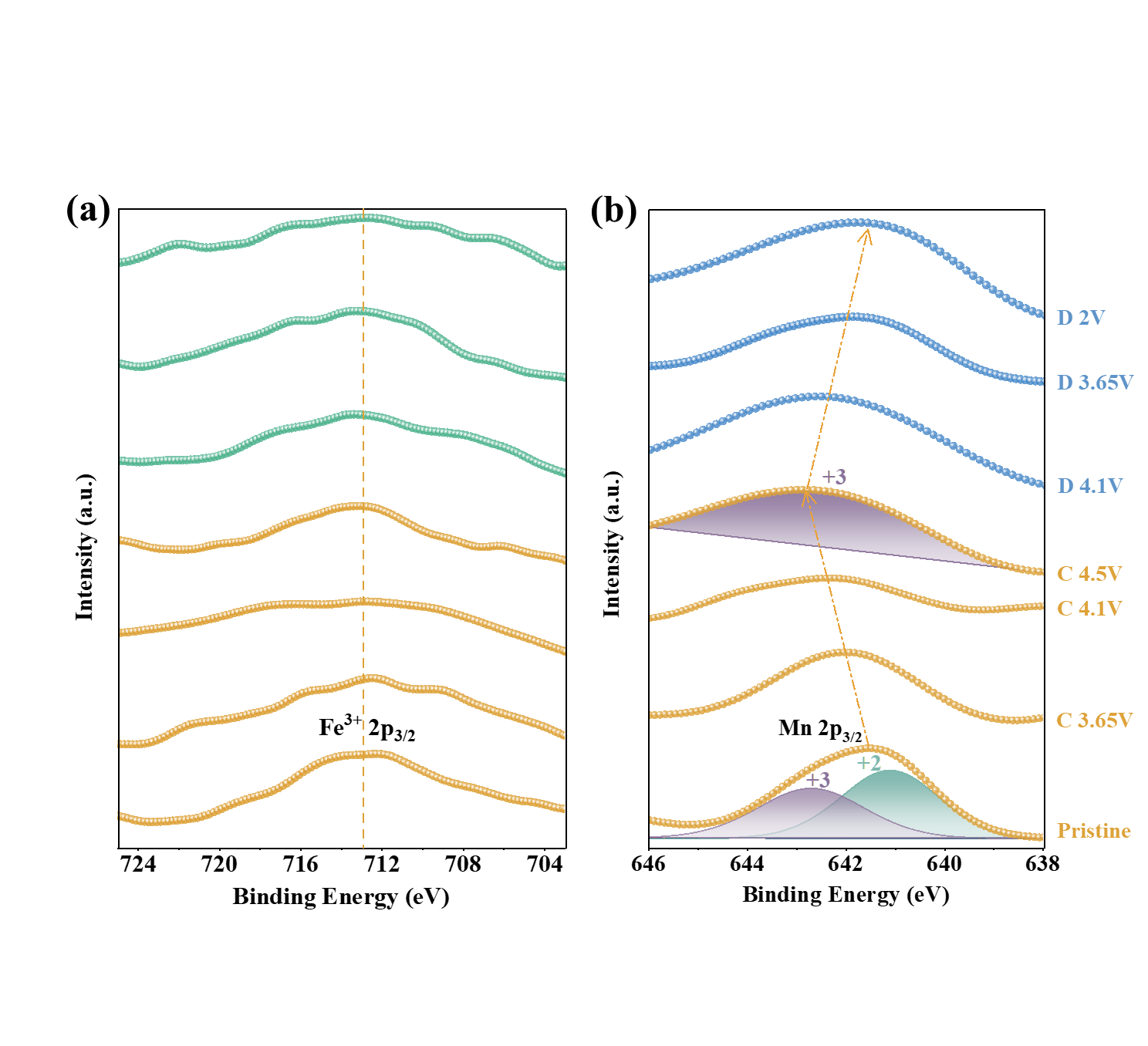


**Fig. S14** Ex-situ XPS spectra: (**a**) Fe 2p_3/2_ and (**b**) Mn 2P_3/2_ of FM-NVPF at different discharging/charging stages


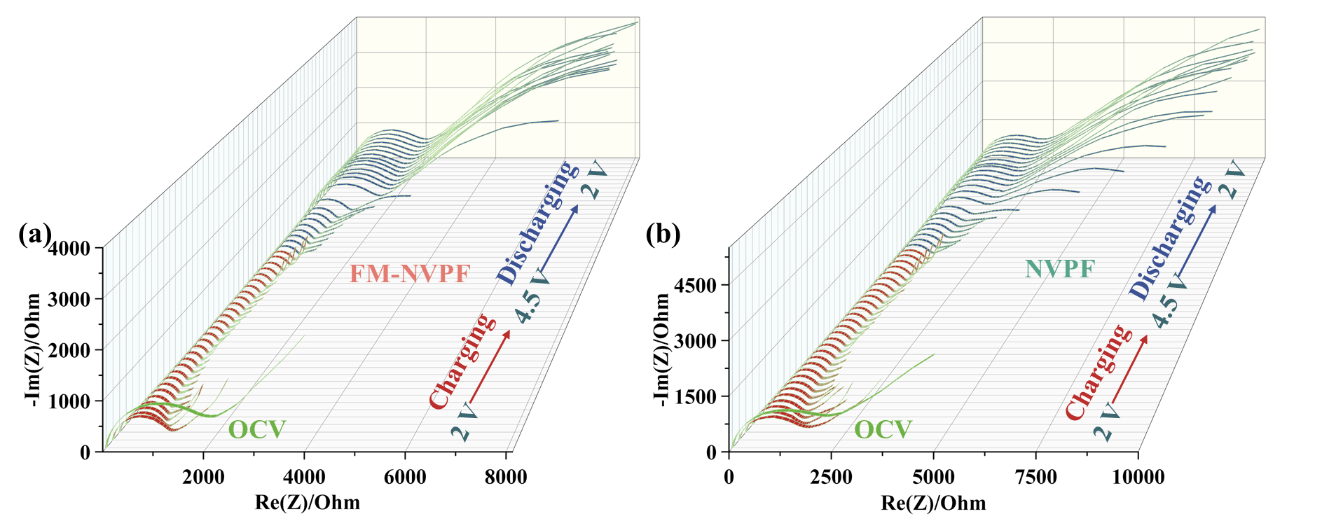


**Fig. S15** In-situ EIS spectra of (a) FM-NVPF and (b) NVPF


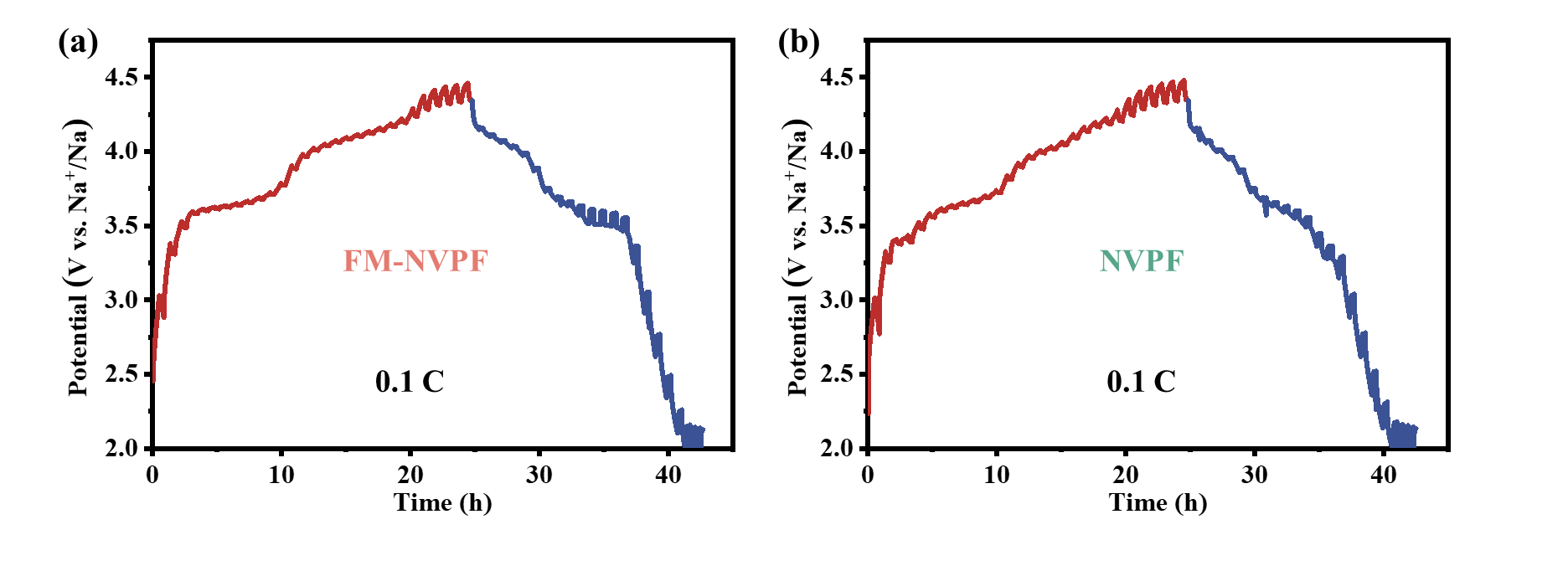


**Fig. S16** The corresponding GCD curves of in-situ EIS spectra for (**a**) FM-NVPF and (**b**) NVPF


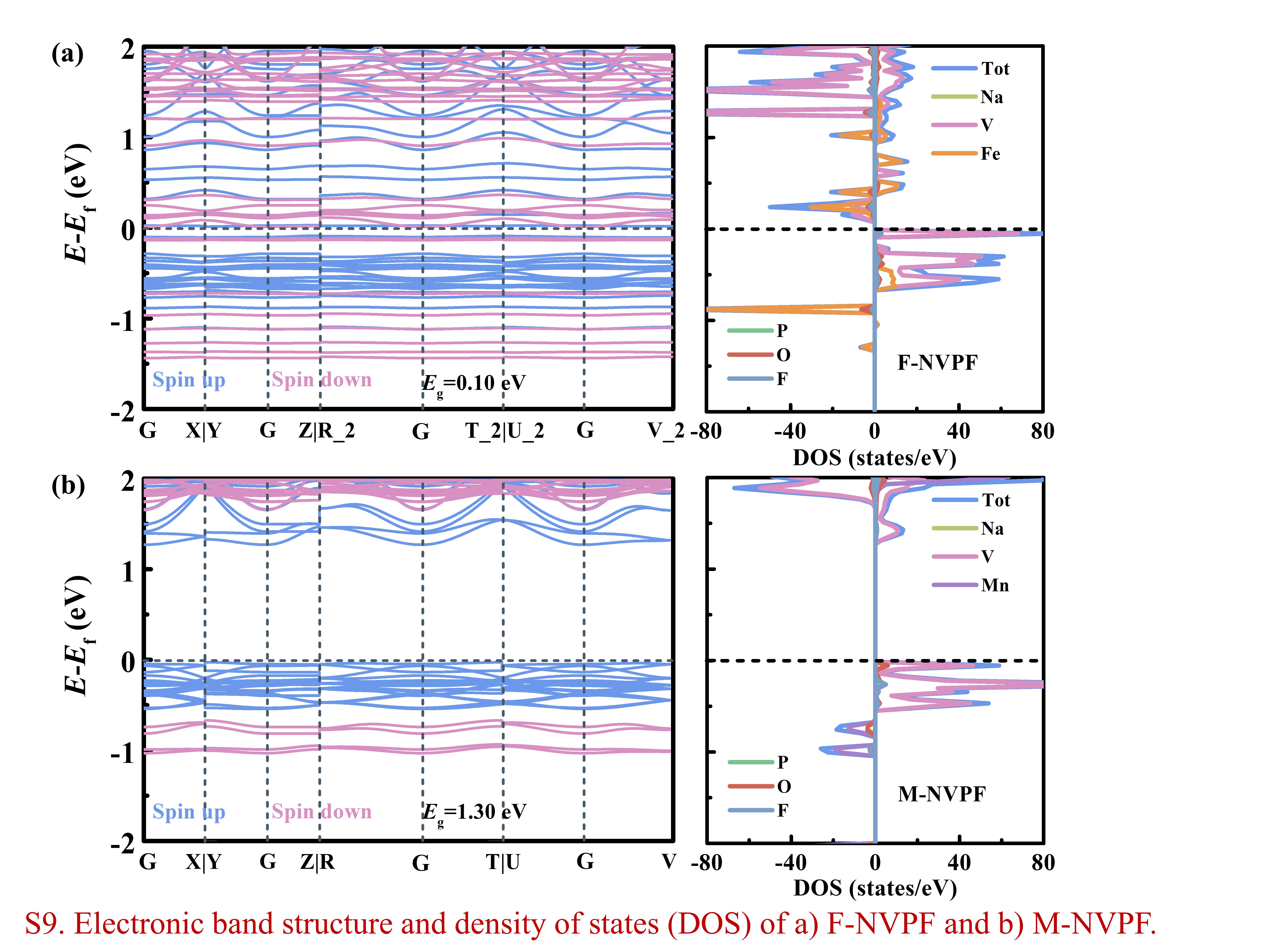


**Fig. S17** Electronic band structure and density of states (DOS) of (**a**) F-NVPF and (**b**) M-NVPF

**
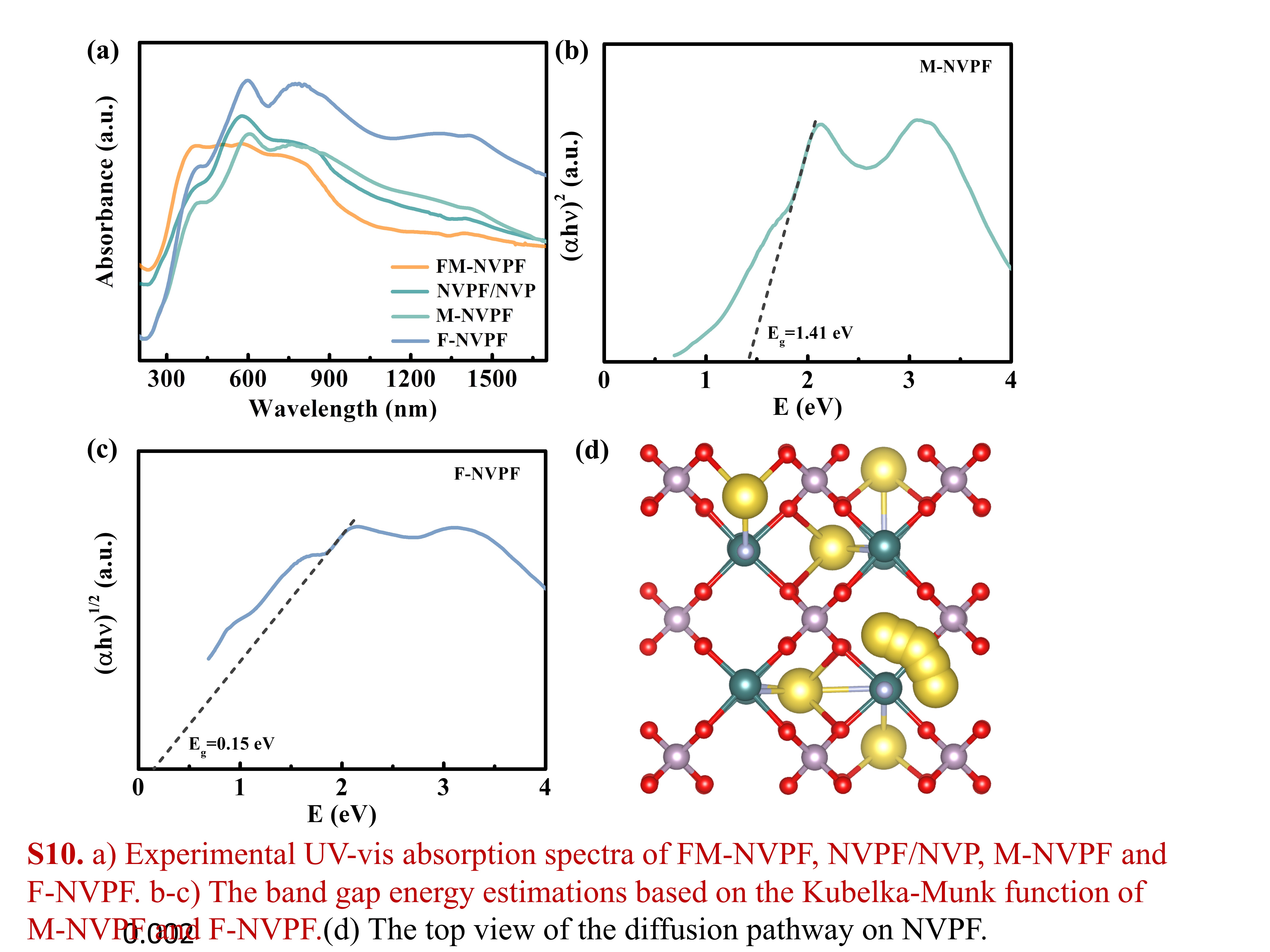
**

**Fig. S18** (**a**) Experimental UV-vis absorption spectra of FM-NVPF, NVPF/NVP, M-NVPF and F-NVPF. The band gap energy estimations based on the Kubelka-Munk function of (**b**) M-NVPF and (**c**) F-NVPF. (**d**) The top view of the diffusion pathway on NVPF


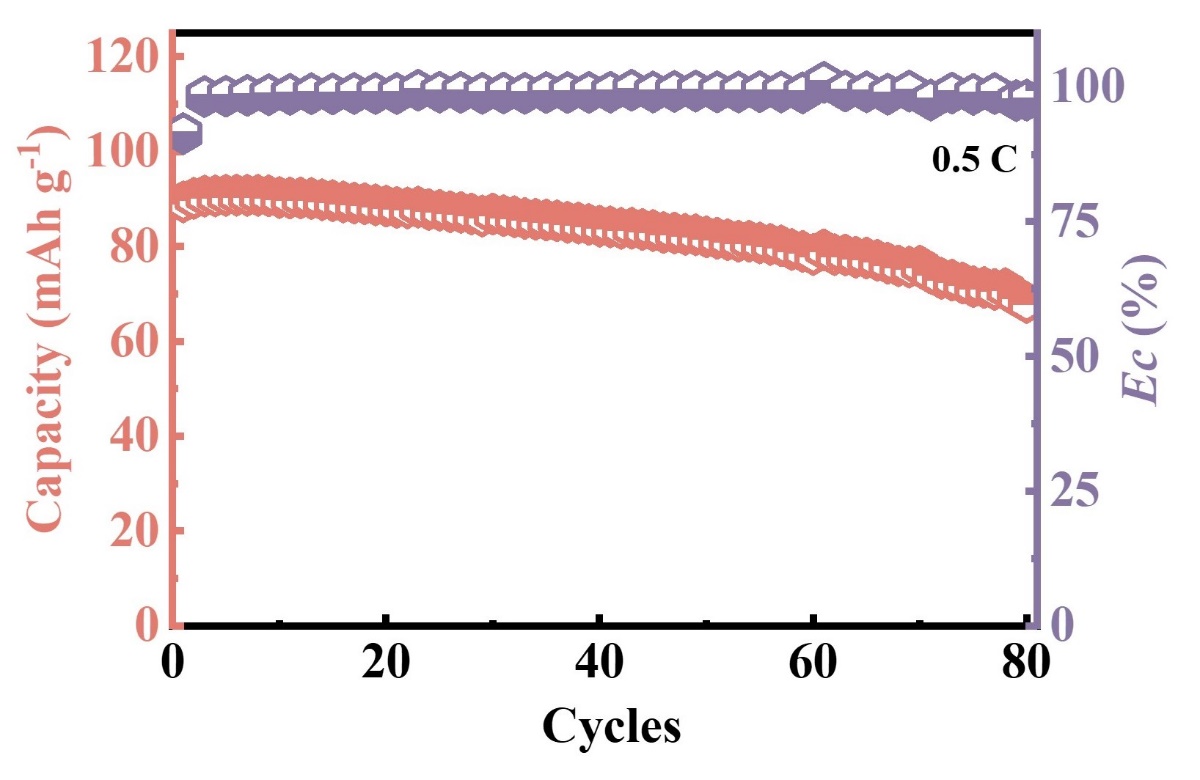


**Fig. S19** Cycling performance of the full cell at 0.5 C

**Table S1** Results of crystal analyses by Rietveld refinements in FM-NVPF with *P42/mnm* space group.

| Sample | *a* (Å) | *b* (Å) | *c* (Å) | *α*, *β*, *γ* (°) | *V* (Å^3^) | *R*_wp_^*^ |
| --- | --- | --- | --- | --- | --- | --- |
| FM-NVPF | 9.083 | 9.083 | 10.732 | 90 | 885.351 | 0.045 |

^*^ *R*_wp_: weighted profile residual.

**Table S2** Atomic parameters of FM-NVPF with *P42/mnm* space group

| Atom | Symmetry | *x* | *y* | *Z* |
| --- | --- | --- | --- | --- |
| Na1 | 1 | 0.518682 | 0.251159 | 0 |
| Na2 | 1 | 0.809553 | 0.056031 | 0 |
| V1 | 1 | 0.246595 | 0.246595 | 0.189981 |
| Mn1 | 1 | 0.246595 | 0.246595 | 0.189981 |
| Fe1 | 1 | 0.246595 | 0.246595 | 0.189981 |
| P1 | 1 | 0 | 0.5 | 0.25 |
| P2 | 1 | 0 | 0 | 0.256577 |
| O1 | 1 | 0.093197 | 0.398951 | 0.160302 |
| O2 | 1 | 0.092283 | 0.092283 | 0.154008 |
| O3 | 1 | 0.455386 | 0.320556 | 0.600490 |
| F1 | 1 | 0.245171 | 245171 | 0 |
| F2 | 1 | 0.242644 | 0.242644 | 0.362984 |

**Table S3** Element contents obtained from XPS spectum

| Sample/(At. %) | Na | Fe | Mn | V | P | O | F |
| --- | --- | --- | --- | --- | --- | --- | --- |
| NVPF | 9.46 | - | - | 8.33 | 11.92 | 64.36 | 5.93 |
| FM-NVPF | 7.44 | 1.35 | 1.15 | 5.56 | 11.38 | 65.44 | 7.67 |

**Table S4** EIS parameters obtained by fitting EIS spectra of NVPF, FM-NVPF

| Parameter | *R_s_* (Ω) | *R_ct_* (Ω) | *σ* | *D* (cm^2^/s) |
| --- | --- | --- | --- | --- |
| NVPF | 4.897 | 1648.1 | 2057.9 | 6.56E-15 |
| FM-NVPF | 1.452 | 741.3 | 460.3 | 1.31E-13 |

**Table S5** Comparison of the total energies (*E*_0_) of FM-NVPF with Mn doping at V sites (1-7) at different distances from Fe.

| Sites | *E*_0_ (eV) |
| --- | --- |
| 1 | -505.981 |
| 2 | -505.818 |
| 3 | -504.581 |
| 4 | -504.333 |
| 5 | -504.618 |
| 6 | -502.799 |
| 7 | -505.974 |

**Table S6** The electronic conductivity of materials obtained by the four-point probe

| Samples | Conductivity (mS/cm) |
| --- | --- |
| NVPF | 1.10E-3 |
| FM-NVPF | 1.71E-3 |

**Supplementary References**

1. L. Li, J. Zhao, H. Zhao, Y. Qin, X. Zhuet al., Structure, composition and electrochemical performance analysis of fluorophosphates from different synthetic methods: Is really Na_3_V_2_(PO_4_)_2_F_3_ synthesized? J. Mater. Chem. A. **10**(16), 8877-8886 (2022). <https://doi.org/10.1039/d2ta00565d>
2. C. Xu, J. Zhao, E. Wang, X. Liu, X. Shenet al., A novel nasicon‐typed Na_4_VMn_0.5_Fe_0.5_(PO_4_)_3_ cathode for high‐performance na‐ion batteries. Adv. Energy Mater. **11**(22), 2100729 (2021). <https://doi.org/10.1002/aenm.202100729>
3. X. H. Liu, W. H. Lai, J. Peng, Y. Gao, H. Zhanget al., A nasicon‐typed Na_4_Mn_0.5_Fe_0.5_Al(PO_4_)_3_ cathode for low‐cost and high‐energy sodium‐ion batteries. Carbon Neutralization. **1**(1), 49-58 (2022). <https://doi.org/10.1002/cnl2.6>
4. X. Ou, X. Liang, C. Yang, H. Dai, F. Zhenget al., Mn doped NaV_3_(PO_4_)_3_/C anode with high-rate and long cycle-life for sodium ion batteries. Energy Stor. Mater. **12**, 153-160 (2018). <https://doi.org/10.1016/j.ensm.2017.12.007>
5. J. Liang, S. Luo, D. Pan, P. Xu, F. Zhan et al., Metal organic framework derived coniooh nanorods anchored on carbon cloth as electrodes for asymmetric supercapacitors. Chem. Eng. J. **464**, 142646 (2023). <https://doi.org/10.1016/j.cej.2023.142646>
